# Dynamics of Charge-Density-Wave Puddles in 2*H*‑NbSe_2_

**DOI:** 10.1021/acs.nanolett.6c03967

**Published:** 2026-10-12

**Authors:** Shreya Kumbhakar, Marina Esposito, Anjan Kumar N M, Liwen Feng, Tommaso Confalone, Vasilisa Gerega, Flavia Lo Sardo, Rafiqul Alam, Davide Masarotti, Francesco Tafuri, Thomas Böhm, Mahmoud Abdel-Hafiez, Sushmita Chandra, Claudia Felser, Kornelius Nielsch, Nicola Poccia, Stefan Kaiser, Golam Haider

**Affiliations:** † Institute of Solid State and Materials Physics, 9169 TUD Dresden University of Technology, Dresden D-01062, Germany; ‡ Leibniz Institute for Solid State and Materials Research, Helmholtzstr. 20, Dresden D-01069, Germany; § Department of Physics, University of Naples Federico II, Naples I-80126, Italy; ∥ Institute of Applied Physics, 9169 TUD Dresden University of Technology, Dresden D-01062, Germany; ⊥ Institute of Materials Science, 9169 TUD Dresden University of Technology, Dresden D-01062, Germany; # Department of Electrical Engineering and Information Technology, University of Naples Federico II, Naples I-80126, Italy; 7 Department of Applied Physics and Astronomy, 59105 University of Sharjah, 27272 Sharjah, United Arab Emirates; 8 Max Planck Institute for Chemical Physics of Solids, Dresden D-01187, Germany

**Keywords:** Charge density wave, van der Waals materials, Amplitude modes, Fano coupling, Ultrafast spectroscopy, Raman spectroscopy

## Abstract

Electronic phases in quantum materials are often governed by nanoscale inhomogeneity, where local order develops within spatially confined regions, or *puddles*. A prominent example is an incommensurate charge-density-wave (I-CDW) comprising locally commensurate domains. In 2*H*-NbSe_2_, such an I-CDW state persists alongside lattice anharmonicity and superconductivity, raising fundamental questions about the dynamical stabilization of CDW order in puddles. Here, using Raman scattering, we reveal a strong Fano-coupling between interlayer shear phonons and the CDW amplitude mode. Our time-resolved reflectivity measurement shows a low-frequency ∼0.2 THz coherent overdamped oscillation onsetting within the CDW regime at ∼17 K, indicating a previously unexplored transition. This we identify as a Fano-coupled phonon-CDW hybrid emerging from the collective dynamics of CDW puddles. We establish an overdamped-oscillator framework for capturing such intertwined glassy dynamics. These results highlight how lattice pinning and electronic correlations shape CDW order in layered materials, insight crucial for designing novel van der Waals devices.

## Graphical abstract

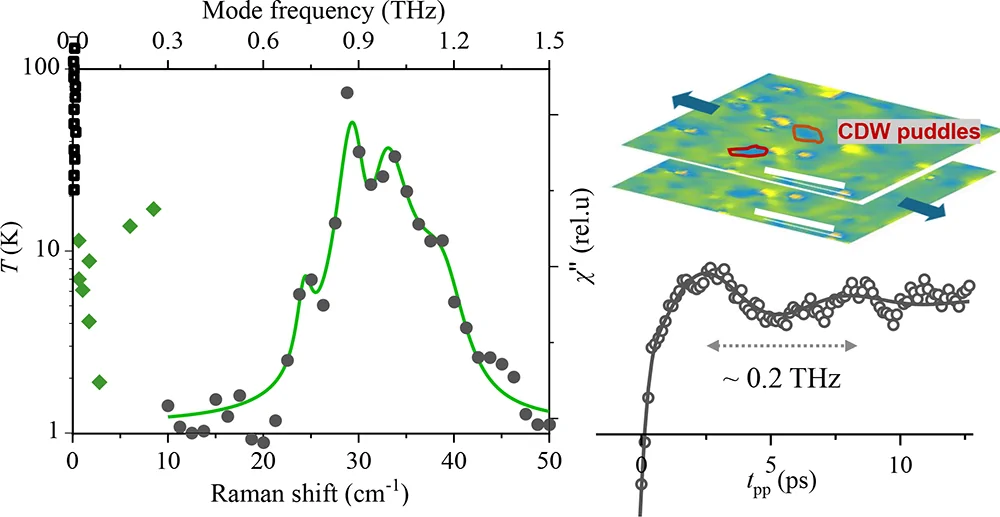

Strongly correlated electron systems, in which lattice, charge, spin, and orbital degrees of freedom interact actively with each other, naturally develop local inhomogeneities or “*puddles*” of distinct types of order^1–12^ , driven by the competition of different phases.^13,14^ Recent experiments^15–17^ suggest that the coexistence of these puddles can generate a multiband or multigap system,^16,18^ where new interference effects emerge from a generalized “Fano” coupling^19^ between the different bands. Importantly, such systems, ranging from ultracold gases to high-*T*_*c*_ cuprates,^20–26^ can be tuned close to a Fano resonance condition through doping, external pressure, internal chemical pressure or ‘microstrain’ in layered materials, magnetic fields, or geometrical control,^16,17,27–29^ where particle–particle correlations and scattering intensities are strongly enhanced. Here, we investigate 2*H*-NbSe_2_, a prominent system where both superconductivity (SC) and unconventional CDW phases coexist at low temperatures.^30–37^ The CDW phase comprises puddles of distinct local CDW commensurations.^7,38,39^ We study the temporal dynamics of the puddles, which remain elusive despite extensive investigation of their spatial distribution.^7,38–41^ The simpler chemical structure of 2*H*-NbSe_2_ among materials with complexity^3^ helps in disentangling the CDW-puddle dynamics from other intertwined orders,^15,35,42–44^ making it a suitable material choice.

The puddle-dynamics can provide unique insights into the formation of the CDW order itself. For example, although phonons soften strongly near the CDW wave vector in 2*H*-NbSe_2_,^34,45^ it remains debated whether phonons alone drive the transition or whether electronic correlations play an essential role. One of the primary anomalies causing this debate is the persistence of CDW-like spectral features above *T*_CDW_ before the onset of phonon-softening or in the absence of substantial lattice distortions. The microscopic character of the pronounced short-range CDW order observed well above *T*_CDW_ ^31,39,40,46^ remains unclear, i.e., whether they represent amplitude vs phase incoherence, domain dynamics, or preformed electronic order. The very nature of CDW in 2*H*-NbSe_2_ is also not understood clearly as to why the CDW wave vector does not coincide with a strong nesting feature of the Fermi surface^47,48^ and is not perfectly commensurate, raising the question of how anharmonic lattice effects,^34,45^ momentum- and orbital-dependent electron–phonon coupling,^48^ and higher-order free-energy terms stabilize a particular triangular pattern of the CDW domains.^7,49,50^

In this study, we perform Raman scattering and ultrafast spectroscopy measurements on bulk-like mechanically exfoliated flakes of 2*H*-NbSe_2_. We find that the CDW amplitude mode exhibits a strong Fano line shape that captures the coupling between the interlayer shear mode and the CDW mode. Time-resolved reflectivity of the crystals reveals an overdamped oscillation that is intertwined with the electronic response, tracking both CDW and SC orders, setting in at *T*_CDW_ ∼ 28 K and *T*_*c*_ ∼ 7 K, respectively. The divergence of the electron relaxation time in the ultrafast response, together with the low frequency of the oscillation at ∼14−17 K within the CDW phase, establishes an onset of a coherent collective dynamics of the CDW puddles, which is *glassy* in nature. A correlation between the Fano coupling parameter and the oscillation frequency suggests that a competition between different CDW commensuration orders and an early onset of SC fluctuations is reflected in the puddle dynamics.

Figure 1(a) shows the Raman susceptibility, χ″, measured in bulk flakes of 2*H*-NbSe_2_ mechanically exfoliated inside a glovebox and loaded in situ for environment-protected Raman measurements. (See left inset of Figure 1(a) and Supporting Information Sec I, II, and III for more details on material growth, electrical characterization, and Raman measurements). We focus on the low-frequency spectral region up to 60 cm^–1^. We observe a peak at 29 cm^–1^, corresponding to the interlayer shear vibration.^51^ We identify another peak at 35 cm^–1^ and a broad shoulder at 40 cm^–1^, indicated by the arrow. At these frequencies, the CDW amplitude mode^37,52–57^ has been reported (see Supporting Information IV for more discussion on CDW amplitude modes in 2*H*-NbSe_2_). A weak additional feature is also visible near 25 cm^–1^ on the low-frequency side of the shear mode, which we attribute to a split component of the interlayer shear vibration (see discussion of Figure 3(b)). These features reveal a coupling between the interlayer phonon and the CDW order that is accurately described by a Fano line shape (green solid line),^58–60^ sketched in the right inset of Figure 1(a), that we capture with the following expression.

1 $$l\left(\omega \right)=\pi r\left(\omega \right)\frac{{A_{ph}}^{2}{\left(q+\epsilon \right)}^{2}}{\left(1+\epsilon^{2}\right)}$$

where

$$\epsilon =\frac{\omega -\omega_{CDW}-\nu^{2}R\left(\omega \right)}{\pi \nu^{2}r\left(\omega \right)+\gamma_{CDW}},q=\frac{\nu \eta +\nu^{2}R\left(\omega \right)}{\pi \nu^{2}r\left(\omega \right)+\gamma_{CDW}}$$

ω is the Raman-frequency shift, and ν characterizes the coupling strength between the two modes. See Supporting Information Sec IV for further details of the Fano line shape analysis. Such a Fano coupling is intimately connected to the presence of puddles of local order in the system,^15–18^ in this case, the CDW puddles,^7,38,39^ motivating us to investigate its dynamics. Figure 1(b) shows the time-resolved ultrafast optical reflectivity changes, Δ*R*/*R*_0_ at 2 K in single crystals of 2*H*-NbSe_2_. (See Supporting Information Sec V for details on the pump–probe experiments.) We observe and accurately model the response as a convolution of (i) a fast rise in Δ*R*/*R*_0_ close to zero pump–probe delay (*t*_*pp*_ = 0), followed by (ii) an overdamped oscillation before approaching (iii) a slow decay as follows.

2 $$\begin{array}{ll} \frac{\Delta R}{R_{0}} & =A\left(erf\left(\frac{t-t_{0}}{t_{rise}}\right)+1\right)\times \left(e^{-t/\tau_{decay}}+B\right)\\ & ,\times \left(e^{-\gamma t}cos\left(2\pi ft+\Phi \right)+C\right) \end{array}$$

τ_*decay*_ represents the relaxation time of the decay dynamics, and *f* is the frequency of the overdamped oscillation. The intrinsic frequency of oscillation, *f*_0_ is $f_{0}=\sqrt{f^{2}+{\left(\frac{\gamma }{2\pi }\right)}^{2}}$ , where γ is the damping factor. (See Section VI in Supporting Information for further details on the fitting.) We find this overdamped oscillation has a low frequency of ∼0.2 THz, compared to the CDW amplitude mode ∼1 THz.^37,41,56,61^ In the following, we are going to identify this as a new collective mode in the system emerging from the CDW puddles. The characteristic puddle dimensions reported by STM are typically ∼10 nm,^7,38–41^ far below the optical spot sizes employed in the present Raman and ultrafast experiments. Our measurements therefore probe the collective dynamics driven by a macroscopic coherent ensemble of puddles.

**Figure 1:**
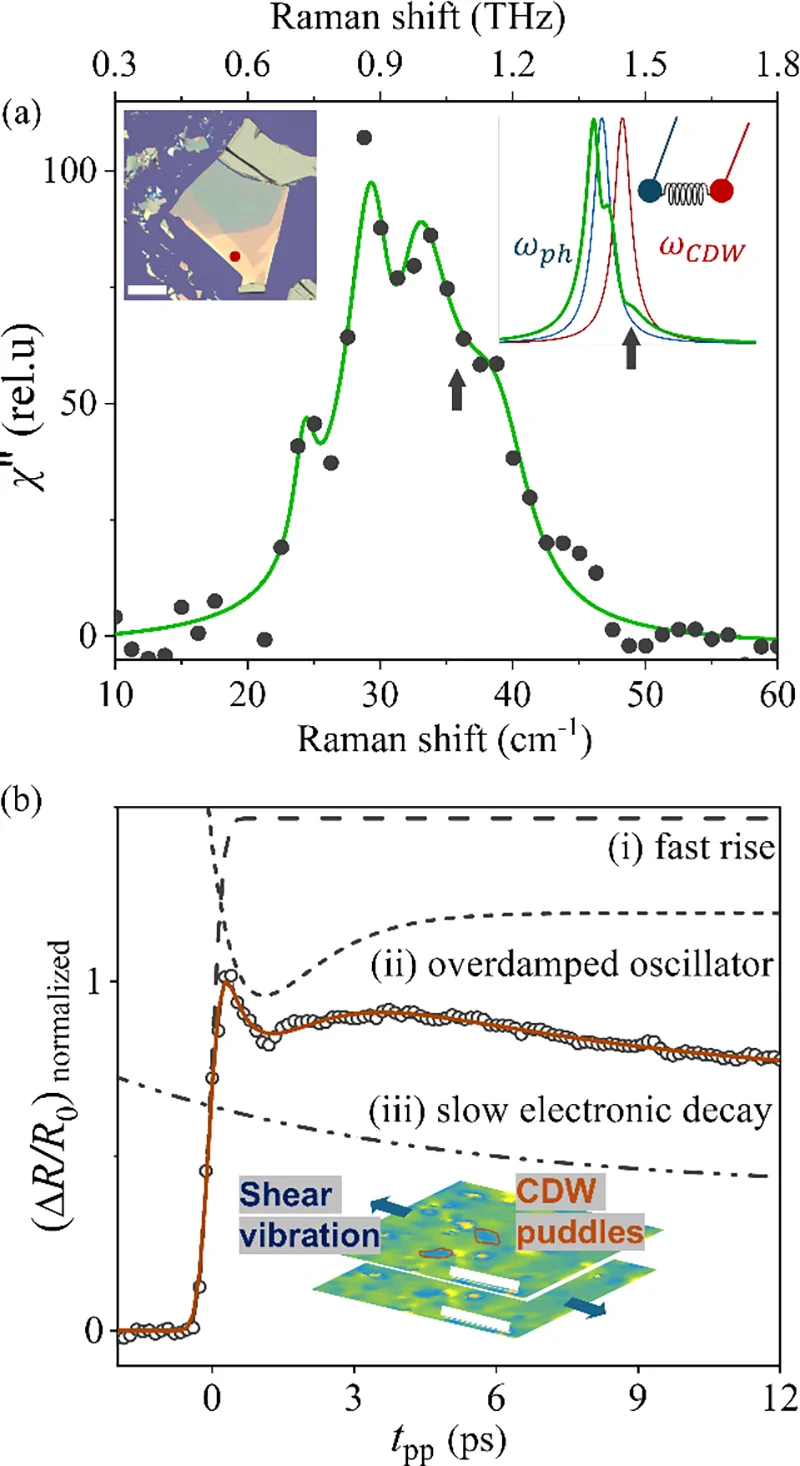
**Fano-coupled phonon-CDW response.** (a) Raman susceptibility, χ″ measured on a mechanically exfoliated bulk flake (thickness ∼60 nm) of 2*H*-NbSe_2_ on Si/SiO_2_ substrate at 14 K is shown. The arrow points to a broad shoulder at ∼40 cm^–1^. The green solid line shows a fit to the data using a Fano resonance model, as sketched in the right inset of Figure 1a. Left inset: Optical image of the flake of 2*H*-NbSe_2_, with the red dot indicating the measurement spot. Right inset: Fano coupling between two discrete oscillators, modeled by Lorentzian lineshapes (blue and red solid curves), leads to the Breit-Wigner Fano line shape (green solid line). The black arrow represents the shoulder, which is analogous to the data in (a). (b) Time-resolved reflectivity change (Δ*R*/*R*_0_), normalized with respect to its peak value, of 2*H*-NbSe_2_ single crystals measured at *T* ∼ 2 K with 80 μJ/cm^2^ of 1.2 eV pump fluence is shown. *R*_0_ is the static probe reflectivity and *t*_*pp*_ is the pump–probe delay. The data is fitted by a convolution of three distinct components, which are shown separately by the dashed, dotted, and dashed-dotted lines, respectively and discussed further in the main text. Inset: The schematic depicts CDW puddles (represented by regions marked with red solid lines). The scale bar corresponds to 10 nm length scales. The blue arrows indicate the interlayer shear vibration that couples with the CDW. The color maps in the square sheets, indicating the layers, are CDW modulations measured via STM experiments reproduced from ref 38.

In Figure 2(a), we show the *T*-dependent ultrafast response in 2*H*-NbSe_2_ from 100 K down to 2 K (see Supporting Information Sec V for detailed *T*-dependence). We observe a noticeable drop in the overall amplitude of the Δ*R*/*R*_0_ above *T*_*c*_ ∼ 6 – 7 K, and even more significant reduction above ∼13.7 K. Furthermore, above 17 K, Δ*R*/*R*_0_ changes sign. We will show in further analysis that 17 K is also the temperature below which the overdamped oscillation (Figure 1(b)) sets in. This we are going to identify as the onset of a glassy dynamics. The largest negative response in Δ*R*/*R*_0_ is reached close to *T*_CDW_ ∼ 28 K. The transition temperatures for SC and CDW, *T*_*c*_ and *T*_CDW_, respectively, have been extracted from the transport characterization in Figure 2(b), with blue- and beige-shaded regions marking the corresponding phases in Figure 2(b)-2(e). These changes in the ultrafast response have been quantitatively captured by the long-lived reflectivity change, Δ*R*/*R*_0_ at *t*_*pp*_ ∼ 13 ps, as shown in Figure 2(c). Figure 2(a) also shows the overdamped oscillations being more prominent at the lowest *T*s below *T*_*c*_ and again close to 17 K, with distinct oscillation frequencies. Analyzing Δ*R*/*R*_0_ of Figure 2(a) with the model discussed in Figure 1(b) (eq 2), Figures 2(d) and 2(e) show the decay time (τ_*decay*_), and, the intrinsic oscillation frequency (*f*_0_) respectively. Note that a conventional multichannel exponential decay model fails to capture the oscillatory features of the glassy dynamics. See Supporting Information Sec VI for more details.

**Figure 2:**
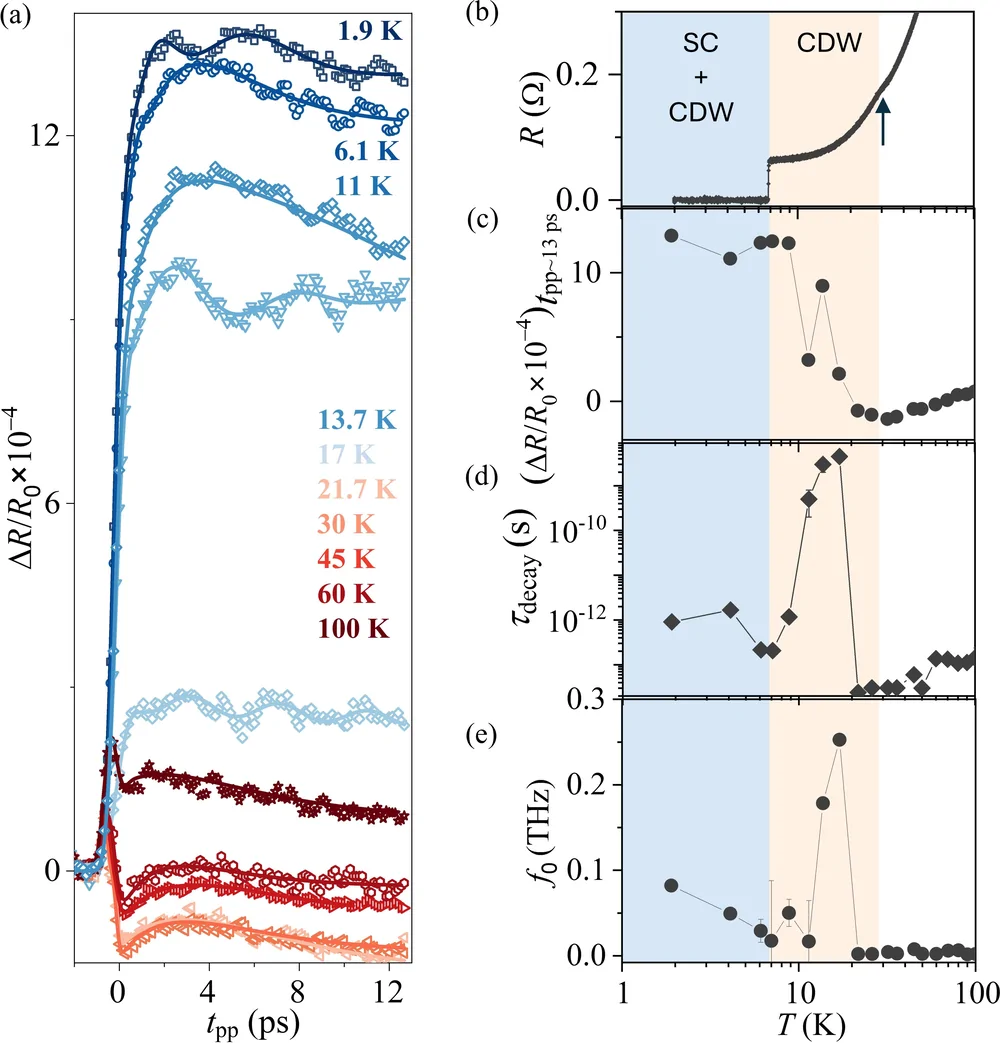
**Temperature-dependent ultrafast dynamics in bulk 2*H*-NbSe**_**2**_ **.** (a) The time-resolved reflectivity, Δ*R*/*R*_0_ is shown at different temperatures (*T*s) from 1.9 K-100 K. The open points are the measured data, and the solid lines are the respective fits using the model discussed in Figure 1(b). (b) Representative *T*-dependent four-probe resistance, *R*, of the 2*H*-NbSe_2_ crystals is shown. *R* drops to nearly zero resistance ( ≈1 nΩ) at *T*_*c*_ ∼ 7 K, indicating the superconducting transition. The data show a kink at the CDW transition temperature, *T*_CDW_ ∼ 28 K, marked by the solid arrow. The blue and beige regions are marked based on these transition temperatures. (c)-(e) show the *T*-dependence of the long-lived reflectivity, i.e. Δ*R*/*R*_0_ at *t*_*pp*_ ∼ 13 ps, the relaxation time, τ_decay_, and overdamped oscillation frequency, *f*_0_, estimated from the fits, respectively.

As the system is cooled to low *T*s, τ_decay_ (in Figure 2(d)) shows a significant increase first at 17 K, and then another rise at *T*_*c*_. The divergence of τ_decay_ is generally connected to a quasiparticle recombination bottleneck effect (e.g., Rothwarf–Taylor bottleneck) associated with gap opening, or from critical slowing down of collective order parameter dynamics near the phase transition.^62,63^ The increase in τ_decay_ close to *T*_*c*_ is consistent with the opening of a SC gap.^62,64,65^ However, the significant enhancement of τ_decay_ at 14–17 K has not been discussed before. Interestingly, τ_decay_ has no sharp change at *T*_CDW_, similar to a previous report.^36^ This is probably because the CDW gap has been observed to be present until room *T*, though incoherent in nature,^46^ with the coherence setting in only at *T*_CDW_.^31^ On the other hand, the diverging behavior of τ_decay_ at 14 – 17 K, where no electronic gap opening has been reported, implies a critical slowing down of electrons from the onset of a glassy dynamics. The values of *τ*_decay_ essentially span the hierarchy of relaxation scales, which has been established independently by Anikin et al.^66^ using broadband pump–probe spectroscopy of 2*H*-NbSe_2_. Their global analysis involving a multiexponential decay model resolves characteristic times of approximately 100 fs, 1 ps, 100 ps and 1 ns. These are associated, respectively, with electron–electron thermalization, electron–phonon relaxation, anharmonic phonon–phonon relaxation and heat dissipation. We note that in the convoluted dynamics, *τ*_decay_ should not be interpreted as the lifetime of one fixed microscopic channel over the entire *T*-range, but the dominant relaxation pathway of the itinerant electronic subsystem. (For details, see Supporting Information Sec VI). Here, the change at 17 K indicates the crossover from a fast IRF-limited electron–electron channel to thermal dissipation and an anharmonic phonon–phonon channel due to the onset of coupled lattice-CDW dynamics.

We observe *f*_0_ (in Figure 2(e)) to be nearly zero above 17 K. At 17 K, it increases sharply to ∼0.25 THz. Below 14 K, we again observe a softening of *f*_0_, down to ∼0.05 THz. Its value remains almost constant until 7 K, close to *T*_*c*_, below which it shows a monotonic increase. The softening of *f*_0_ to almost zero at higher *T*s indicates that the oscillation does not emerge from a simple phononic mode (like coherent phonons), but rather a *new* collective order in the system, which in this case is the CDW order. The low-frequency of the oscillation ( ∼0.2−0.25 THz) further indicates that this is not driven purely by the (fast) CDW amplitude mode (at 40 cm^–1^ ≃ 1.2 THz, as can also be seen from the Raman response in Figure 1(a)) but rather by the (slower) fluctuations of CDW puddles. Also, a recent THz-STM study of 2*H*-NbSe_2_ reported CDW-specific collective dynamics on a similar sub-THz frequency scale, including a mode near 0.15 THz, attributed to spatially heterogeneous, defect-pinned CDW phase dynamics.^41^

We make two key observations here. First, the coherent oscillations set in at 17 K, in the same *T*-window as *τ*_decay_ diverges, supporting the onset of a *glassy* dynamics of CDW-puddles. Glassy dynamics often arise from electronic frustration and/or disorder.^67^ In this case, we believe a strong competition between different CDW commensurations promotes this.^68–70^ Second, the frequency softens below 14 K (to ∼0.05 THz) and increases monotonically below *T*_*c*_. This softening may be related to the onset of SC fluctuations above *T*_*c*_ that affect the puddle dynamics.^5,57,71,72^

To further characterize this complex interplay of phases, we have carried out *T*-dependent Raman measurements, as shown in Figure 3(a). (See Supporting Information Sec III for detailed *T*-dependence.) First, we observe a noticeable reduction in the overall intensity of the spectra above 17 K. This is quantitatively captured by the *T*-dependence of χ″ at 29 cm^–1^ and 35 cm^–1^ (associated generally with the interlayer shear vibration and CDW amplitude modes, respectively) shown in the inset. Second, the features at 35 cm^–1^ and 40 cm^–1^, which we attribute to a ph-CDW Fano coupling effect (discussed in Figure 1(a)), have nontrivial *T*-dependences. Although a peak at 35 cm^–1^ can be identified up to 100 K, the broad shoulder centered at 40 cm^–1^ can be clearly resolved only at lower temperatures (below *T* ∼ 50 K, as we show later). We also note from the inset the nearly constant magnitudes of χ″ with decreasing *T* below *T* ∼ 50 K until 17 K, indicating changes in the Fano coupling behavior at these temperatures. To better understand this, we plot the Raman spectra at two representative temperatures, *T* ∼ 94 and 32 K in the top and bottom panels of Figure 3(b). At *T* ∼ 94 K, the contribution from three distinct Lorentzians captures the data accurately. We note the persistence of the peak at 35 cm^–1^ above *T*_CDW_ is consistent with the observation of the CDW gap even until higher *T*s.^46^ Furthermore, we believe that the peak at 25 cm^–1^ is a splitting (of ≈4 cm^–1^) from the doubly degenerate E_2*g*_ peak at 29 cm^–1^ of the interlayer shear vibration due to strain induced by CDW fluctuations and is hence intimately tied to the CDW amplitude mode. This component persists at lower temperatures. There it is weaker and superposed on the steeply rising low-frequency side of the Fano-coupled response, so it appears as a kink rather than a separate peak (Figure 1(a)). It is included as an additional Lorentzian in the low-temperature Fano fits. Though a splitting of E_2*g*_ peaks from strain has been observed experimentally and predicted theoretically in intercalated NbSe_2_ layers^73^, the same has not been observed on bulk 2*H*-NbSe_2_ layers. At lower temperatures (e.g., at 32 K in the bottom panel of Figure 3(b)), we clearly observe the presence of a broad shoulder at 40 cm^–1^, that is accurately modeled by a Fano resonance between the phonon and CDW amplitude modes (See Supporting Information Sec IV for fit details).

**Figure 3:**
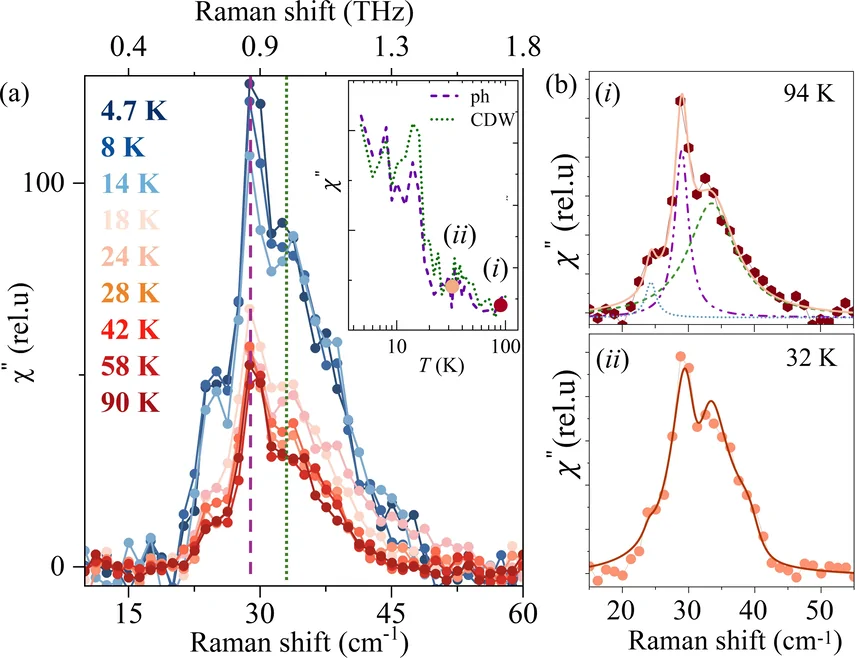
**Temperature dependence of the Raman susceptibility of bulk 2*H*-NbSe**_**2**_ **.** (a) Raman susceptibility (χ″) is measured at different *T*s on bulk flakes of NbSe_2_. The inset plots the value of χ″ at the frequencies marked by the dashed and dotted lines. (b) χ″ is plotted at two distinct *T* ∼ 32 and 94 K. The solid lines represent fits to the data. At 94 K, the data is fitted with additive contributions from three distinct Lorentzian lineshapes, depicted by the dashed lines, which represent the interlayer shear phonon peak at ∼29 cm^–1^, the CDW amplitude mode at ∼35 cm^–1^, and another peak at 25 cm^–1^, that is identified with a split E_2*g*_ peak of the interlayer shear vibration. The data at 32 K is fitted by a Fano line shape, as we discuss further in the text.

Figure 4 plots the *T*-dependence of the Fano coupling parameter, ν, extracted from the Raman response along with *f*_0_. We find a strong fluctuating onset below 50 K, that starts showing a monotonic increase below *T*_CDW_ ∼ 28 K. At 17 K, ν reaches a maximum value, below which it decreases again, yielding a stable plateau from ∼14 K onward to the lowest measurable temperatures. We note that these temperatures do not manifest in the phase characterization from transport (shown by blue and beige regions). Thus, our data capture transitions marking the onset of novel dynamics. (See Supporting Information Sections IV, VI, VII, VIII for additional experimental support.) The Fano coupling is intimately related to phase separation or phase coexistence in a system^16–18^ , which in this case is the CDW puddles. From the onset of ν below 50 K (red shaded region), we conclude that an incoherent puddle regime begins above *T*_CDW_, reminiscent of the dynamical charge-density fluctuations reported well above the charge-order onset in cuprates^44^. A collective coherent motion only sets in at 17 K, captured by the increase of *f*_0_ where ν also attains its maximum strength, likely from the competing CDW orders. This indicates that local response from the puddles, represented by ν, captures the emerging glassy collective dynamics at ∼17 K. With further decrease in *T*, ν stabilizes below ∼14 K where *f*_0_ decreases as well. We could interpret this as a possible onset of SC above *T*_*c*_ (as also proposed in ref 57), that impacts the CDW order and commensuration^35,50^ and is thus manifested in the puddle dynamics.

**Figure 4:**
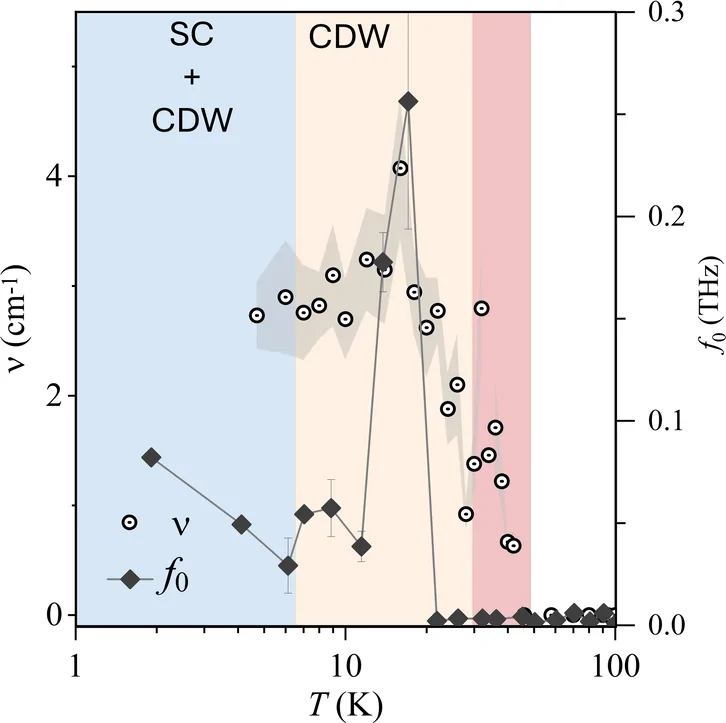
**Temperature-dependence of the Fano coupling factor** **ν, estimated from the fits of the Raman susceptibility, χ″ at different *T*s is plotted on the left-axis.** The gray-shaded region represents the fitting error. The right-axis plots the overdamped oscillation frequency, *f*_0_, obtained from the ultrafast response. Blue and beige shaded regions are marked based on the superconducting and CDW transition temperatures, characterized from electrical transport in Figure 2(b). The red region marks the onset of ν as discussed further in the text.

In summary, both ultrafast and Raman measurements reveal the onset of a new collective dynamics in 2*H*-NbSe_2_ that is driven by the local CDW domains, or puddles. Locally, this manifests as a Fano coupling between the interlayer shear vibration and CDW amplitude mode in the Raman spectra, which directly corroborates the dynamics measured in the ultrafast response. Such a coupling sheds insight into multiple longstanding questions about the CDW phase in NbSe_2_. First, it explains why CDW amplitude mode oscillations corresponding to 40 cm^–1^ could not be clearly observed in the ultrafast response before. Second, it suggests an enhanced coherence of the CDW phase with decreasing interlayer shear strain, providing a possible route for significant enhancement in *T*_CDW_ from the bulk to the monolayer.^52^ Third, the Fano coupling can aid in CDW incommensuration in NbSe_2_, which remains an open question.^34,45,47,48^ Fourth, the microscopic fluctuations observed above *T*_CDW_ ^31,39,40,46^ likely represent preformed electronic order and phase incoherence, as we see signatures of the CDW amplitude mode even until 100 K. Our results establish how local dynamics in puddles shape the global response in a material. This can be used as a recipe to engineer novel functionalities in quantum materials.

## Supplementary Material



## Notes

Material growth, electrical transport measurements, temperature-dependent Raman response, Fano analysis, temperature-dependent ultrafast response, analysis of pump–probe measurements, high-frequency Raman response in 2*H*-NbSe_2_, additional specific heat and THz-THG measurements on 2*H*-NbSe_2_ crystals (PDF)
