# Supplementary material for: Dynamics of Charge-Density-Wave Puddles in 2H‑NbSe2
Source: Nano Lett. 2026 Oct 12;26(41):14376–83. doi: 10.1021/acs.nanolett.6c03967 (PMC13693501; doi:10.1021/acs.nanolett.6c03967)
Supplement: Supplementary file 1 [file nl6c03967_si_001.pdf]

Supporting Information: Dynamics of Charge-Density-Wave *puddles* in $2H$-$NbSe_2$

Shreya Kumbhakar[1,2,⋆], Marina Esposito[2,3,⋆], Anjan Kumar N M[1,⋆], Liwen Feng[1], Tommaso Confalone[2,4], Vasilisa Gerega[2,4], Flavia Lo Sardo[2,5], Rafiqul Alam[2], Davide Masarotti[6], Francesco Tafuri[3], Thomas Böhm[2], Mahmoud Abdel-Hafiez[7], Sushmita Chandra[8], Claudia Felser[8], Kornelius Nielsch[2,5], Nicola Poccia[2,3,†], Stefan Kaiser[1,†], and Golam Haider[2,†]

[1] *Institute of Solid State and Materials Physics, TUD Dresden University of Technology, Dresden, D-01062, Germany*

[2] *Leibniz Institute for Solid State and Materials Research, Helmholtzstr. 20, Dresden, D-01069, Germany*

[3] *Department of Physics, University of Naples Federico II, Naples, I-80126, Italy*

[4] *Institute of Applied Physics, TUD Dresden University of Technology, Dresden, D-01062, Germany*

[5] *Institute of Materials Science, TUD Dresden University of Technology, Dresden, D-01062, Germany*

[6] *Department of Electrical Engineering and Information Technology, University of Naples Federico II, Naples, I-80126, Italy*

[7] *Department of Applied Physics and Astronomy, University of Sharjah, 27272 Sharjah, United Arab Emirates*

[8] *Max Planck Institute for Chemical Physics of Solids, Dresden, D-01187, Germany*

⋆ *equal contribution and*

† *g.haider@ifw-dresden.de, stefan.kaiser@tu-dresden.de, nicola.poccia@unina.it*

I. MATERIAL GROWTH

High-quality single crystals of $2H$-$NbSe_2$ were grown using the conventional chemical vapour transport method. Initially, stoichiometric amounts of niobium powder (Alfa Aesar, 99.99%) and selenium powder (Sigma-Aldrich, 99.98%) were thoroughly mixed and sealed under vacuum in a quartz ampoule with iodine (~ 3 mg cm^{-3}) as the transport agent. The sealed ampoule was placed in a dual-zone furnace with a temperature gradient of 800 °C (source zone) and 720 °C (growth zone) and maintained for a period of 15 days. After slowly cooling to room temperature, shiny layered $2H$-$NbSe_2$ single crystals with typical lateral dimensions of a few millimetres were obtained.

II. ELECTRICAL TRANSPORT MEASUREMENTS

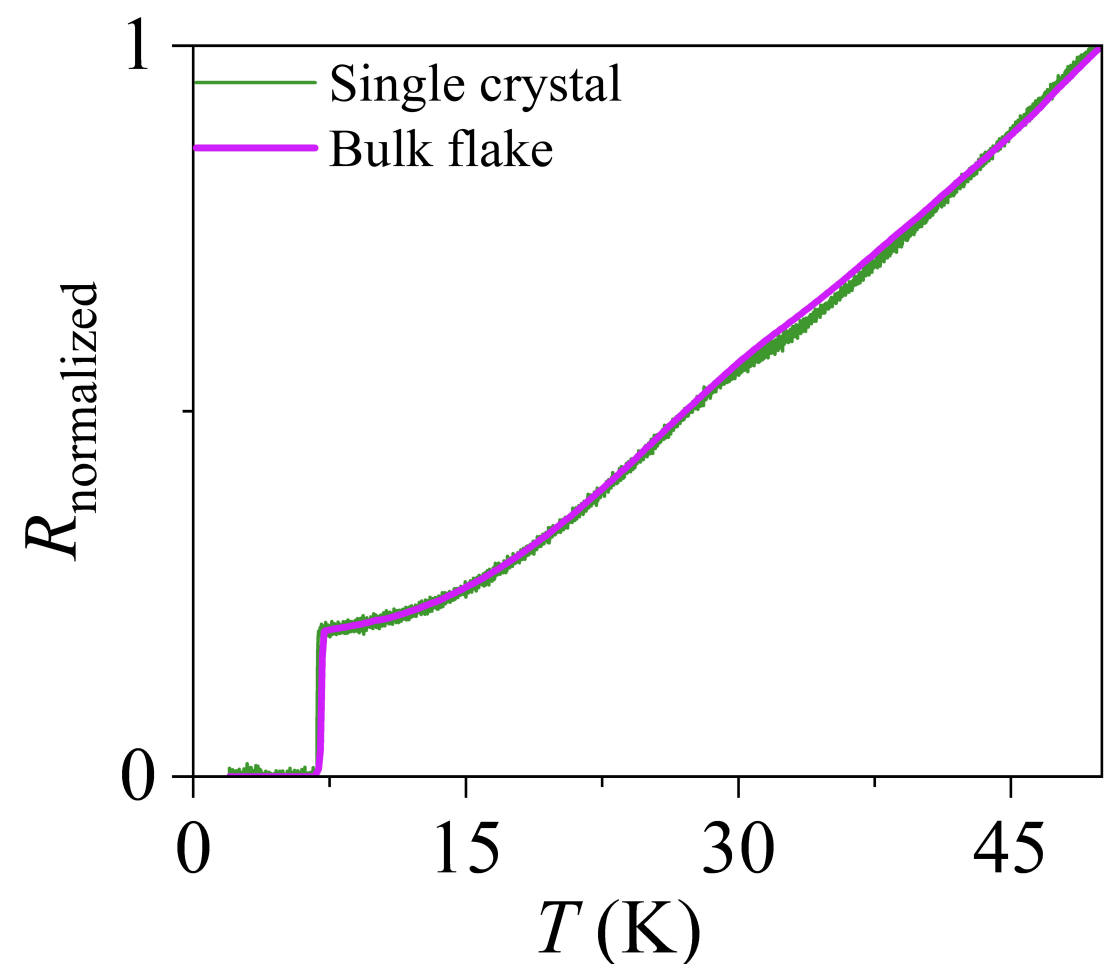

FIG. S1. Four-probe resistance of a $2H$-$NbSe_2$ single crystal and of an exfoliated bulk flake on a Si/SiO_2 substrate. The superconducting and CDW transitions can be identified at $\sim$7 K and $\sim$28 K, respectively.

III. TEMPERATURE-DEPENDENT RAMAN RESPONSE

Raman measurements were carried out in a backscattering configuration using a WITec Alpha 300 confocal Raman microscope equipped with a 50× objective (NA = 0.9), enabling a spot size of 1 micron with a continuous-wave polarized 532 nm laser. For the measurements carried out in this work, we did not implement any polarizer in the detector path. High

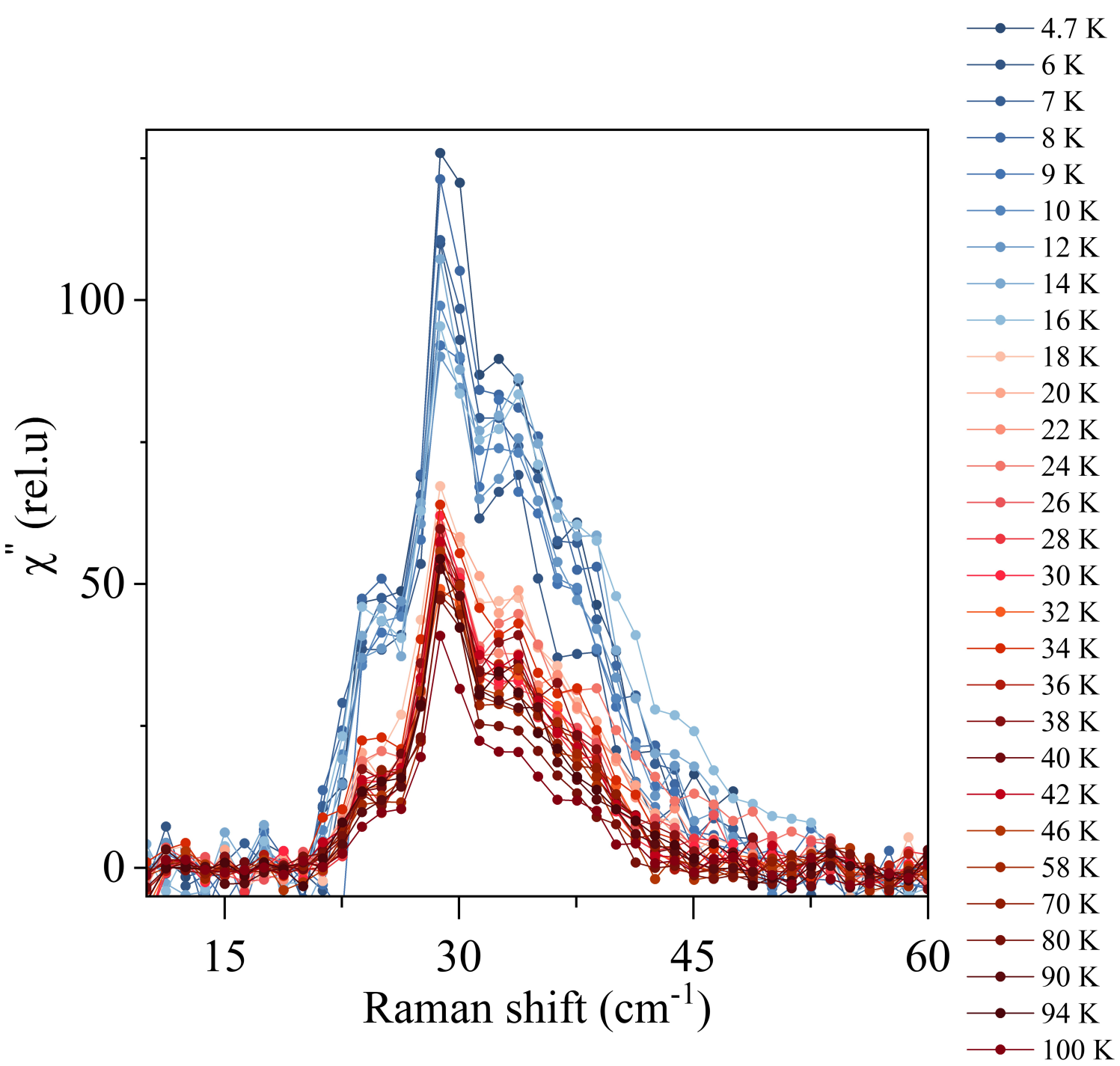

FIG. S2. **Temperature-dependent Raman susceptibility**. Raman susceptibility (χ'') measured on bulk-like (thickness∼ 60 nm) exfoliated flakes of $NbSe_2$ at temperatures varying from 4.7 K to 100 K is shown in the low-frequency spectral range up to 60 cm^{-1}.

spectral resolution was achieved using a diffraction grating with 1800 lines mm^{-1}, providing a resolution of approximately 1.2 cm^{-1}. A 532 nm RayShield filter enabled the detection of low-frequency Raman modes down to 10 cm^{-1} on both the Stokes and Anti-Stokes sides of the spectrum. The laser power at the sample was monitored with a calibrated power meter and set to 1 mW for single-point spectra. This power was confirmed to ensure that the average heating is less than 1 K (60 s per spectrum, averaging over 10 accumulations). See also the discussion below and Section VII for T-estimation and T-dependence of the high-frequency phonon peaks to assess laser-induced heating effects. Cryogenic measurements from 300 K to 3 K were carried out using a continuous-flow cryostat Microstat HiRes from Oxford Instruments.

$NbSe_2$ flakes were exfoliated from the single crystals, grown as discussed above, on cleaned Si/SiO_2 substrates inside an Argon-filled glovebox and loaded in situ in the cryostat for Raman measurements. This ensured pristine surfaces of the flakes were protected from environmental degradation. For the results presented in this manuscript, we have focused on bulk flakes (thickness ∼ 60 nm comprising about 100 atomic layers), ruling out substrate-related effects. We also show the electrical transport characteristics of both the single crystals and exfoliated thick flakes in Sec II of SI.

Fig. S2 shows the low-frequency Raman response for different temperatures. After subtracting a smooth background, the Raman susceptibility was obtained from the measured Stokes intensity by correcting for the Bose-Einstein thermal factor [1],

$$\chi''(\omega) \propto \frac{I(\omega)}{1+n(\omega,T)}, \qquad n(\omega,T) = \frac{1}{e^{\hbar\omega/k_B T}-1}.$$

The spectra have then been fitted with a Fano lineshape as we show in Eq. [1] of the manuscript and discuss further in Section IV.

We estimated the laser-induced heating effects to be less than 1 K. Laser-induced

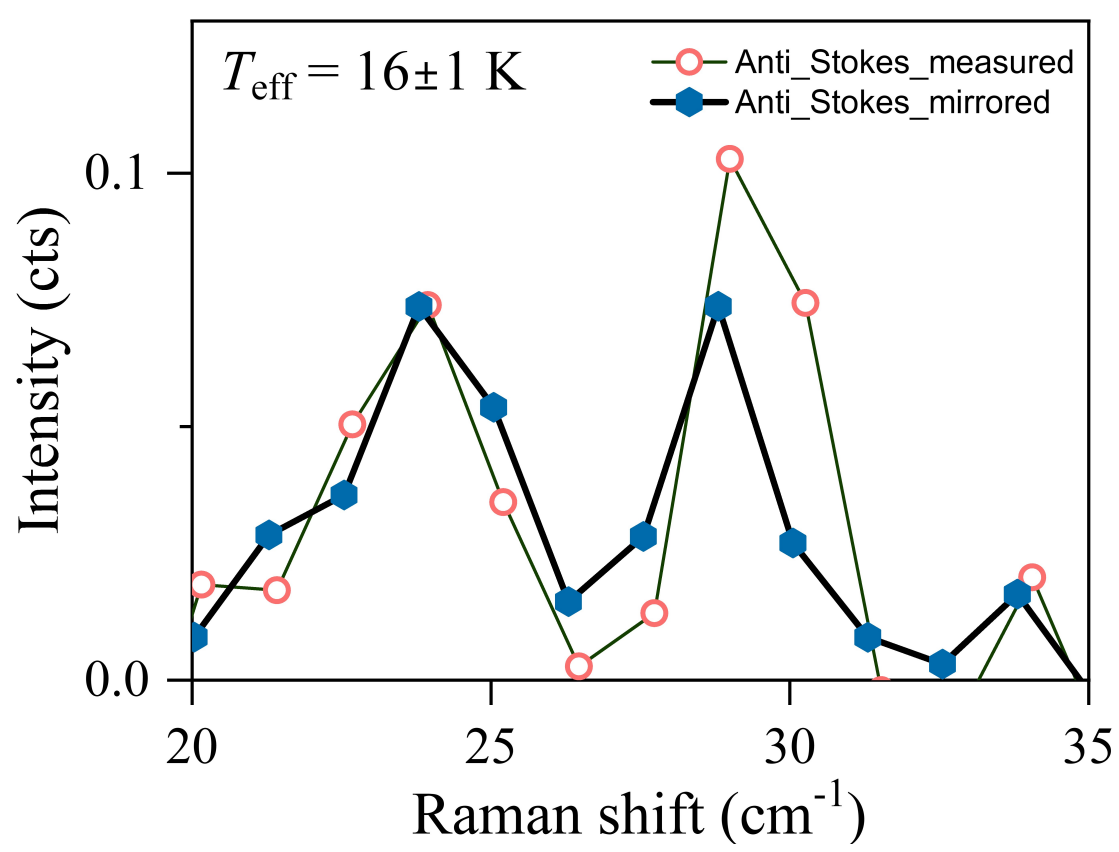

FIG. S3. Measured Anti-Stokes spectrum and Anti-Stokes spectrum simulated from the Stokes intensity using Eq. [1] are plotted.

heating is most commonly assessed through the effective temperature extracted from the Stokes/Anti-Stokes intensity ratio. However, in the present case, the low-energy response originates from a Fano-coupled phonon-CDW mode, for which the relation between the measured intensity and the thermal population is not well understood in the literature. Consequently, applying a conventional Stokes/Anti-Stokes analysis directly to the hybrid mode may not yield a reliable estimate of the lattice temperature. To circumvent this issue, we instead consider the split mode near 25 cm^{-1}, which is well described by a Lorentzian lineshape in our Raman analysis [See discussion of Fig. 3 in the manuscript] and therefore exhibits predominantly phononic character. Using the intensity ratio of the Stokes and Anti-Stokes components (following Eq. 1) of this mode after excluding the ph-CDW response, we estimate the effective temperature of the sample.

$$\frac{I_{AS}(\Delta E)}{I_S(\Delta E)} = \frac{(E+\Delta E)^4}{(E-\Delta E)^4}\exp(-\frac{\Delta E}{k_B T}) \tag{1}$$

Here, E is the photon energy. We show in Fig. S3 the measured Anti-Stokes intensity and the estimated Anti-Stokes intensity from the Stokes data at 16 K using the expression above [2, 3].

We focus on this temperature because it represents the most critical temperature scale identified in the manuscript, where we claim the onset of the coupled collective dynamics. In addition, near this temperature range, the split mode near 25 cm^{-1} can be clearly separated from the ph–CDW response once the Fano contribution is subtracted, allowing a reliable determination of the effective temperature from the Stokes/Anti-Stokes intensity ratio. We find that the measured Anti-Stokes response is reproduced by the mirrored Stokes spectrum

using an effective temperature of $T_{\text{eff}} \approx 16 \pm 1$ K. Thus, within experimental uncertainty, the effective temperature agrees with the bath temperature.

IV. FANO ANALYSIS

To accurately reproduce the interference phenomena observed in the Raman spectrum, we use a model based on a Fano-type resonance describing the coupling between two discrete excitations with finite damping [4–9].

To derive the interference formula, we first define the unperturbed system, which consists of a ground state $|g\rangle$, a discrete excited state $|d\rangle$ with excitation energy E_d, and a second excited state $|b\rangle$ characterized by a broader spectral response and centred at energy E_b.

Instead of solving the secular equation for this system explicitly in terms of its eigenstates, the Green's function $G(z) = (z-H)^{-1}$ can be introduced, where z is an analytic continuation of energy, E and H is the Hamiltonian of the coupled system. The physical Raman intensity is obtained from the imaginary part of the retarded Raman susceptibility $\chi(z)$ evaluated at $z = E + i0^+$.

The starting point is the expression for the Raman intensity obtained from Fermi's golden rule, which can be written in terms of the imaginary part of an effective response function for the coupled system [4]. The resulting Raman spectrum $l(E)$ is:

$$l(E) = \mathrm{Im}\left[\frac{A_d^2(E_b - z) - 2\nu A_b A_d + A_b^2(E_d - z)}{(E_b - z)(E_d - z) - \nu^2}\right];$$

Here, A_d and A_b represent the Raman transition amplitudes from the ground state to the sharp discrete excitation $|d\rangle$ and to the broader excitation $|b\rangle$, respectively. The parameter ν describes the coupling between the two excited states, which gives rise to the hybridization and quantum interference between the corresponding Raman scattering channels.

The effect of the broader excitation is incorporated in the Green's function $G(z)$. In this formulation the inverse propagator $(E_b - z)$ is replaced by $G(z)^{-1}$ where

$$G(z) = \sum_b \frac{1}{E_b - z}.$$

The imaginary and real parts of $G(z)$ represent energy-dependent spectral functions:

$$r(E) = \pi^{-1}\,\mathrm{Im}\,[G(E + i0^+)], \qquad R(E) = -\mathrm{Re}\,[G(E + i0^+)].$$

Following the derivation by Klein [4], the resulting energy-dependent Raman intensity can be written as:

$$l(E) = \pi r(E) A_b^2 \frac{(E_d - E - \nu\eta)^2}{(E_d - E + \nu^2 R(E))^2 + \pi^2\nu^4 r(E)^2}. \tag{2}$$

where $\eta = A_d/A_b$ denotes the ratio of the Raman transition amplitudes. $R(E)$ accounts for the energy renormalization of the resonance, while $r(E)$ determines the coupling-induced linewidth. Including a finite intrinsic linewidth γ_d of the sharp excitation in addition to the coupling-induced broadening leads to the modified Fano-type lineshape used for fitting the experimental data [4–9].

$$\begin{aligned} l(E) =& \frac{\pi r(E) A_b^2 \left[(E - E_d + \nu\eta)^2 + \gamma_d\left(\nu^2\pi r(E) + \gamma_d\right)\right]}{(E - E_d - \nu^2 R(E))^2 + (\nu^2\pi r(E) + \gamma_d)^2} \\ &+ \frac{\gamma_d^2\left(\nu R(E) + \eta\right)^2}{(E - E_d - \nu^2 R(E))^2 + (\nu^2\pi r(E) + \gamma_d)^2} \end{aligned} \tag{3}$$

The total linewidth and the asymmetric line shape thus results from the combined effect of intrinsic damping and interference-induced broadening. Eq. (3) can be written in a form equivalent to that given by Fano [10] namely:

$$l(E) = \pi r(E) A_b^2 (q+\epsilon)^2/(1+\epsilon^2)$$

where

$$\epsilon = [E - E_d - \nu^2 R(E)]/[\pi \nu^2 r(E) + \gamma_d]$$

and

$$q = [\nu\eta + \nu^2 R(E)]/[\pi \nu^2 r(E) + \gamma_d].$$

In this representation, ϵ measures the detuning from the renormalised resonance energy in units of the interaction-induced linewidth, while q quantifies *generalized* Fano-coupling by combining the relative strength and phase of the two interfering Raman amplitudes.
The expression that we have used to fit the Fano lineshape in the measured Raman spectra is as follows.

$$\begin{aligned} l(\omega) =& \frac{A_{\mathrm{ph}}^2 \pi r(\omega) \left[(\omega - \omega_{\mathrm{CDW}} + \nu\eta)^2 + \gamma_{\mathrm{CDW}} \left(\nu^2 \pi r(\omega) + \gamma_{\mathrm{CDW}} \right) \right]}{(\omega - \omega_{\mathrm{CDW}} - \nu^2 R(\omega))^2 + (\nu^2 \pi r(\omega) + \gamma_{\mathrm{CDW}})^2} \\ &+ \frac{A_{\mathrm{ph}}^2 \gamma_{\mathrm{CDW}}^2 \left(\nu R(\omega) + \eta \right)^2}{(\omega - \omega_{\mathrm{CDW}} - \nu^2 R(\omega))^2 + (\nu^2 \pi r(\omega) + \gamma_{\mathrm{CDW}})^2} \end{aligned} \tag{4}$$

where:

$$r(\omega) = \frac{\gamma_{\mathrm{ph}}}{(\omega - \omega_{\mathrm{ph}})^2 + \gamma_{\mathrm{ph}}^2}$$

and $R(\omega)$ is derived as Hilbert transform of $r(\omega)$.

Here, ω is the Raman frequency shift and is the equivalent of energy E defined before. $\omega_{\mathrm{ph}}, \omega_{\mathrm{CDW}}$ are the centre frequencies, $\gamma_{\mathrm{ph}}, \gamma_{\mathrm{CDW}}$ are the broadening or intrinsic damping of the oscillators corresponding to the phonon and the CDW modes, respectively. Furthermore, $\eta = A_{\mathrm{CDW}}/A_{\mathrm{ph}}$ defines the relative oscillator strength of these modes. We note that the labels 'discrete' and 'broad' refer only to the roles of the two states in the formalism above. The assignment of the modes follows from the fitted centre frequencies: the state entering through $r(\omega)$ converges to $\omega_{\mathrm{ph}} \approx 29$ cm^{-1}, matching the interlayer shear phonon, and is therefore assigned to the phonon, while the other state ($\omega_{\mathrm{CDW}} \approx 35$ cm^{-1}) is assigned to the CDW amplitude mode. Although this differs from the conventional Fano picture, where the continuum-like state is the broader one, it is the assignment that consistently reproduces the measured spectra. The Fano coupling then contributes an additional damping, $\nu^2\pi r(\omega)$, to the CDW mode on top of its intrinsic damping γ_{CDW}. In this formulation, the state entering through $G(z)$ acts as a structured reservoir that renormalizes the other excitation. The assignment thus implies that the CDW amplitude mode is dressed by, and borrows Raman weight from, the interlayer shear phonon ($A_{\mathrm{ph}} > A_{\mathrm{CDW}}$, Fig. S4(b)). The larger intrinsic damping γ_{CDW} is consistent with the known damping of the amplitude mode in metallic $2H$-$NbSe_2$, and likely also reflects inhomogeneous broadening from a distribution of local CDW environments across puddles. Consistent with this, γ_{CDW} decreases on cooling through the crossover regime between approximately 17 K and T_{CDW} (Fig. S4(d)), as local CDW correlations strengthen on approach to the coherent regime.

Fig. S4 plots the T-dependence of the parameters extracted from the Fano fits to the Raman response, with (a), (b), (c) and (d) respectively plotting ν, $1/\eta^2$, (ω_{ph}, ω_{CDW}), and (γ_{ph}, γ_{CDW}). The T-dependence of ν in Fig. S4(a), discussed in the main text, captures (i) an early onset of puddles, which we define as an incoherent puddle regime below 50 K, and

(ii) a maximum strength close to 17 K, which marks the onset of a coherent-puddle phase, likely emerging from the competition between different CDW commensuration orders. $1/\eta^2$ (Fig. S4(b)) saturates below $T \sim 17$ K, consistent with a stable coupling between the lattice and the CDW order that leads to the formation of this coherent phase.

The mode frequencies ω_{ph} and ω_{CDW} (Fig. S4(c)) and the damping constants γ_{ph} and γ_{CDW} (Fig. S4(d)) do not show a distinct change at 17 K. Instead, their most pronounced changes occur at higher temperatures, between approximately 17 K and T_{CDW}, with the sharpest variation near 23 K. Both frequencies increase and γ_{CDW} decreases on cooling through this interval. This is consistent with a hardening of the interlayer shear vibration as the CDW puddles become pinned and more strongly interlayer-correlated. The microscopic origin of this behavior is not yet clear. Within our experimental resolution of 1.2 cm^{-1}, we therefore do not take it as evidence for a distinct transition temperature. We instead regard the interval between approximately 17 K and T_{CDW} as a crossover regime in which the coupled phonon-CDW response evolves continuously. The identification of 17 K as the onset of the coherent collective regime is thus based on the correlated anomalies observed across multiple independent observables and experimental probes, namely ν, $1/\eta^2$, τ_{decay}, f_0, t_{onset}, Φ, C, the specific heat, and the THz-THG response.

Finally, we would like to emphasise that the low-energy Raman response in $2H$-$NbSe_2$ remains surprisingly unresolved despite decades of study. A notable aspect of the reported CDW amplitude mode is that it is unusually broad, with linewidths of the order of tens of cm^{-1}, which is itself unexpected for a well-defined collective excitation, for example, in comparison to the Higgs mode identified in the very same material. This broad response has been recognized since the early Raman studies of $2H$-$NbSe_2$, yet its microscopic origin remains debated. While the amplitudon was first identified in Ref. [11], subsequent reports have shown significant variation in both the position and linewidth of the CDW-related feature. Some studies report a broad response centred near 45 cm^{-1} with a linewidth approaching 20 cm^{-1} [11, 12], whereas others identify a feature closer to 35 cm^{-1} [13–15]. Likewise, the relative intensity between the interlayer shear phonon and the CDW-related response varies substantially across the literature. These discrepancies indicate that the low-energy Raman response of $2H$-$NbSe_2$ remains incompletely understood, despite the extensive attention the material has received over the past several decades. Indeed, much of the recent attention has focused on the superconducting Higgs mode and its coupling to the CDW state, focusing primarily on temperatures up to 10 K, whereas a detailed temperature-dependent investigation of the CDW-related Raman response itself has remained largely unexplored. The hybridization between the interlayer shear phonon and the CDW response, which we discuss, explains both the unusually broad lineshape and the apparent variation in the reported peak position between 35 and 45 cm^{-1}. In fact, the presence of a Fano-type interaction between the phonon and CDW response was already pointed out by Mialitsin [7]. What has been missing, however, was a systematic temperature-dependent investigation of this coupling and its relation to the emergence of a distinct collective dynamics. By combining temperature-dependent Raman spectroscopy with ultrafast measurements, the present work provides such a framework and demonstrates that the evolution of the Fano coupling parameter is directly linked to the onset of a new collective response. Within this framework, the observed Raman response is determined not only by the intrinsic CDW excitation but also by the relative spectral weight of the phononic and CDW components. Consequently, modest variations in the relative strengths of these coupled contributions can naturally produce Raman responses that appear centred closer to either 35 or 45 cm^{-1}, thereby providing a unified interpretation of the existing literature.

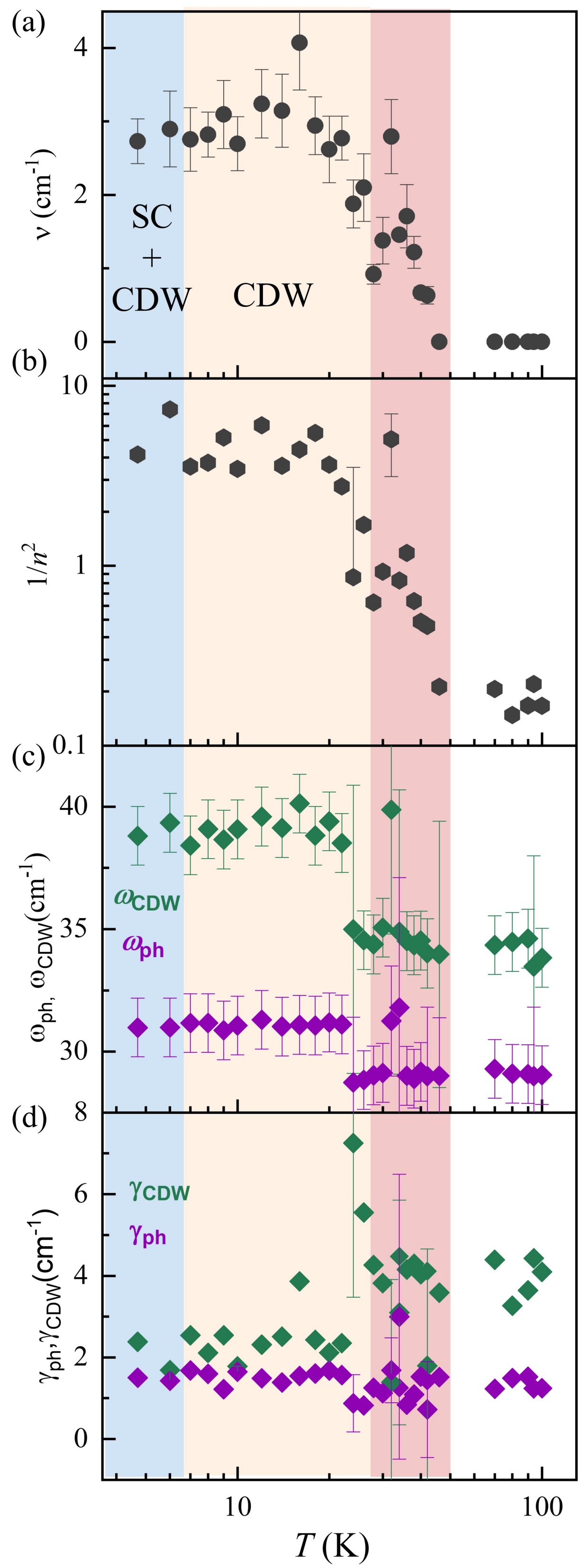

FIG. S4. **Parameters extracted by fitting the Raman response with the Fano lineshape.** (a), (b), (c) and (d) respectively plot the Fano coupling (ν), inverse squared amplitude ratio ($1/\eta^2 = (A_{\rm ph}/A_{\rm CDW})^2$), centre frequencies ($\omega_{\rm ph}, \omega_{\rm CDW}$) and damping ($\gamma_{\rm ph}, \gamma_{\rm CDW}$).

V. TEMPERATURE-DEPENDENT ULTRAFAST RESPONSE

We performed femtosecond nondegenerate pump–probe reflectivity measurements on high-quality single crystals. The ultrafast experiments used 190 fs laser pulses in a reflection setup, with a 1030 nm pump beam and a 680 nm probe beam. The laser pulses were generated by a regenerative amplifier system (Pharos, Light Conversion; 1030 nm central wavelength, 10 kHz repetition rate, 190 fs pulse duration). The output beam was split into two parts. One part was sent to an optical parametric amplifier to produce the 680 nm probe beam, while the 1030 nm beam was used as the pump to excite the sample.

The pump and probe beams were linearly polarized and set perpendicular to each other to reduce coherent artifacts and polarization-related interference effects. The time delay between the pump and probe pulses was controlled using a motorized delay stage. The pump beam was modulated with a mechanical chopper. The resulting pump-induced change in reflectivity ($\Delta R/R_0$) was measured using a photodiode and lock-in detection for improved signal sensitivity.

Temperature-dependent measurements were performed in a continuous-flow helium cryostat (Optistat CF, Oxford Instruments), which provided an inert environment and maintained a clean sample surface during the experiment. The full-width at half maximum (FWHM) spot sizes were about 200 μm for the pump and 100 μm for the probe, ensuring uniform excitation within the measured area. The pump and probe fluences are kept constant at 40 μJ/cm^2 (that is below the threshold for the suppression of the CDW state [16–18]), and 20 μJ/cm^2, respectively, for all temperatures studied in Fig. 2.

VI. ANALYSIS OF PUMP-PROBE MEASUREMENTS

The Fano-coupled ph-CDW order that we present invokes an intertwined dynamics between lattice and charge degrees of freedom. In this description, the transient reflectivity cannot be captured by additive contributions from independent decay channels, as is commonly done in conventional pump-probe studies. The time-resolved reflectivity is instead described by a convolution of different decay dynamics: a fast rise, slow decay, and an overdamped oscillator to fit the transient time-resolved reflectivity as follows.

$$\frac{\Delta R}{R_0} = A(\mathrm{erf}(\frac{t-t_0}{t_{rise}}) + 1) \times (e^{-\frac{t}{\tau_{decay}}} + B) \times (e^{-\gamma t}\cos(2\pi f t + \Phi) + C) \tag{5}$$

Here, t_0 is the time zero of the response and t_{rise} is its rise time. To account for the finite temporal resolution of the experiment, we corrected the fitted t_{rise} for the instrument response function (IRF, cross-correlation of approximately 300 fs) by deconvolution. We denote this IRF-corrected intrinsic rise time as t_{onset}; these are the values reported in Fig. S7(a).

τ_{decay} represents the relaxation time of the decay dynamics.
A constant offset is required to define the electronic baseline associated with each channel. B is an offset to the electronic decay, which physically represents a heating term. C can be interpreted as an incoherent offset to the collective oscillation, without which the entire response would oscillate around zero. As coherence is lost, the oscillatory term decays to zero, whereas the incoherent contribution may remain finite.

f is the frequency of the overdamped oscillation. The intrinsic frequency of oscillation, f_0 is $f_0 = \sqrt{f^2 + (\frac{\gamma}{2\pi})^2}$, where γ is the damping factor.

Fig. S6 plots $\Delta R/R_0$ after deconvolving the measured data with the fast rise and slow decay, using Eq. [5] at a few representative temperatures. We observe an overdamped oscillation, captured by the solid lines, that has approximately a time-period (estimated from

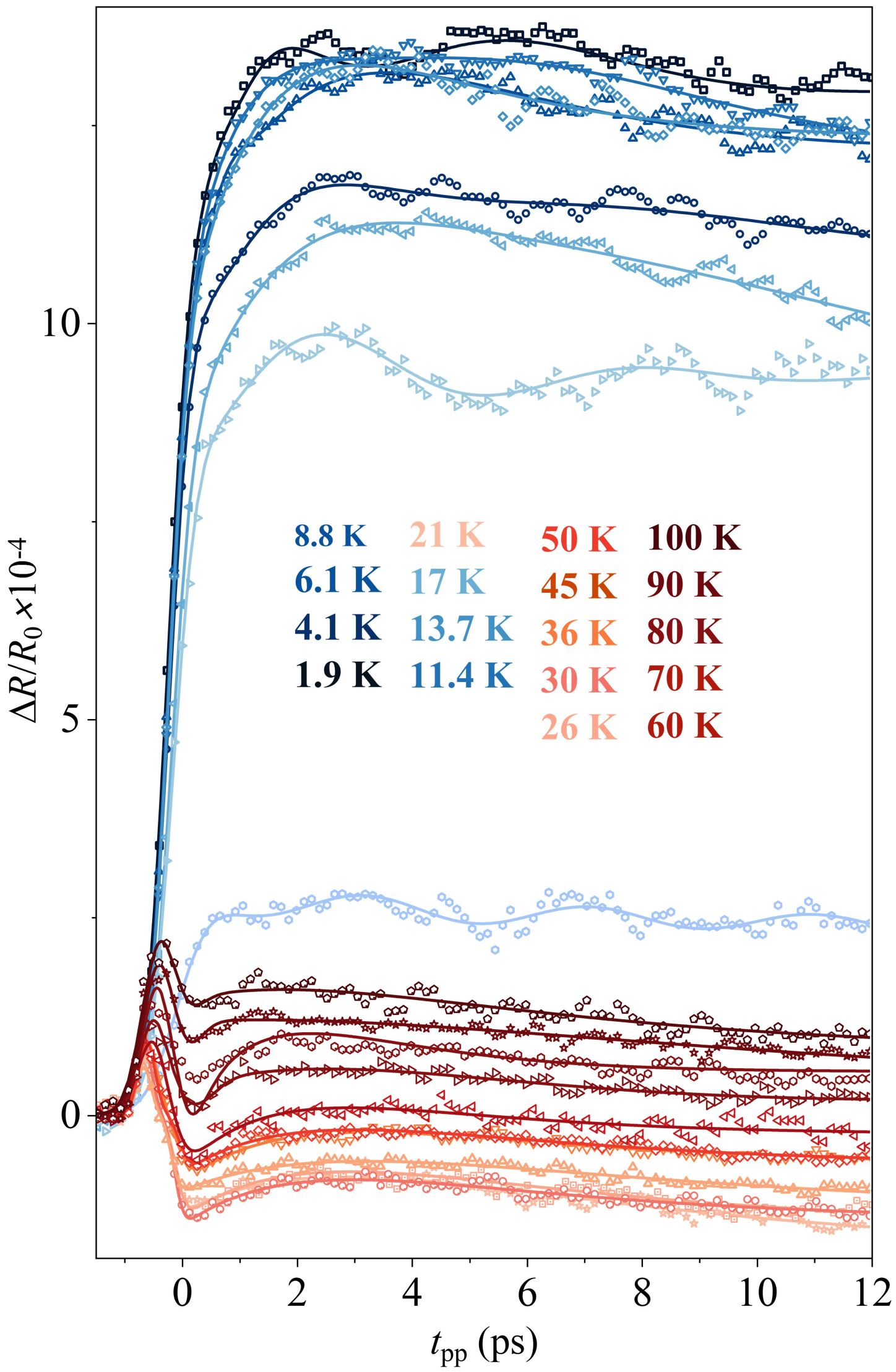

FIG. S5. Time-resolved reflectivity change (ΔR) normalized with the static probe reflectivity, R_0, has been measured at temperatures varying from 1.9 K to 100 K. The open points represent experimental data, and the solid lines show the corresponding fits using Eq. [2] of the main text.

the minimum close to zero time delay to the dip-like feature imposed on the slow decay) of 10 ps at 1.9 K. Close to 14 K, we observe the oscillation to be faster, having an approximate time period of 5 ps, again becoming slower ($\geq$ 12 ps) at 26 K. This matches overall the T-dependence of the overdamped oscillation, f_0, that we show in Fig. 2(e) of the main text as well as in Fig. S13(d). Fig. S7(a) plots the onset time of the ultrafast response, t_{onset} at different temperatures. t_{onset} increases across $\sim$ 17 K at lower temperatures, indicating a slowing down of the onset of the ultrafast response, which is quantitatively similar to [18], where this is attributed to CDW order-parameter suppression with higher temperature. However, a noticeable increase at $T \sim$ 17 K (and not at T_{CDW}), as well as a drop at $\sim$ 45 K, cannot be entirely explained by this. We believe that below 50 K, an *incoherent* puddle dynamics sets in, which shows a coherent oscillation only below $\sim$ 17 K. The order-parameter suppression is likely more effective in the incoherent puddle phase, whereas in the coherent

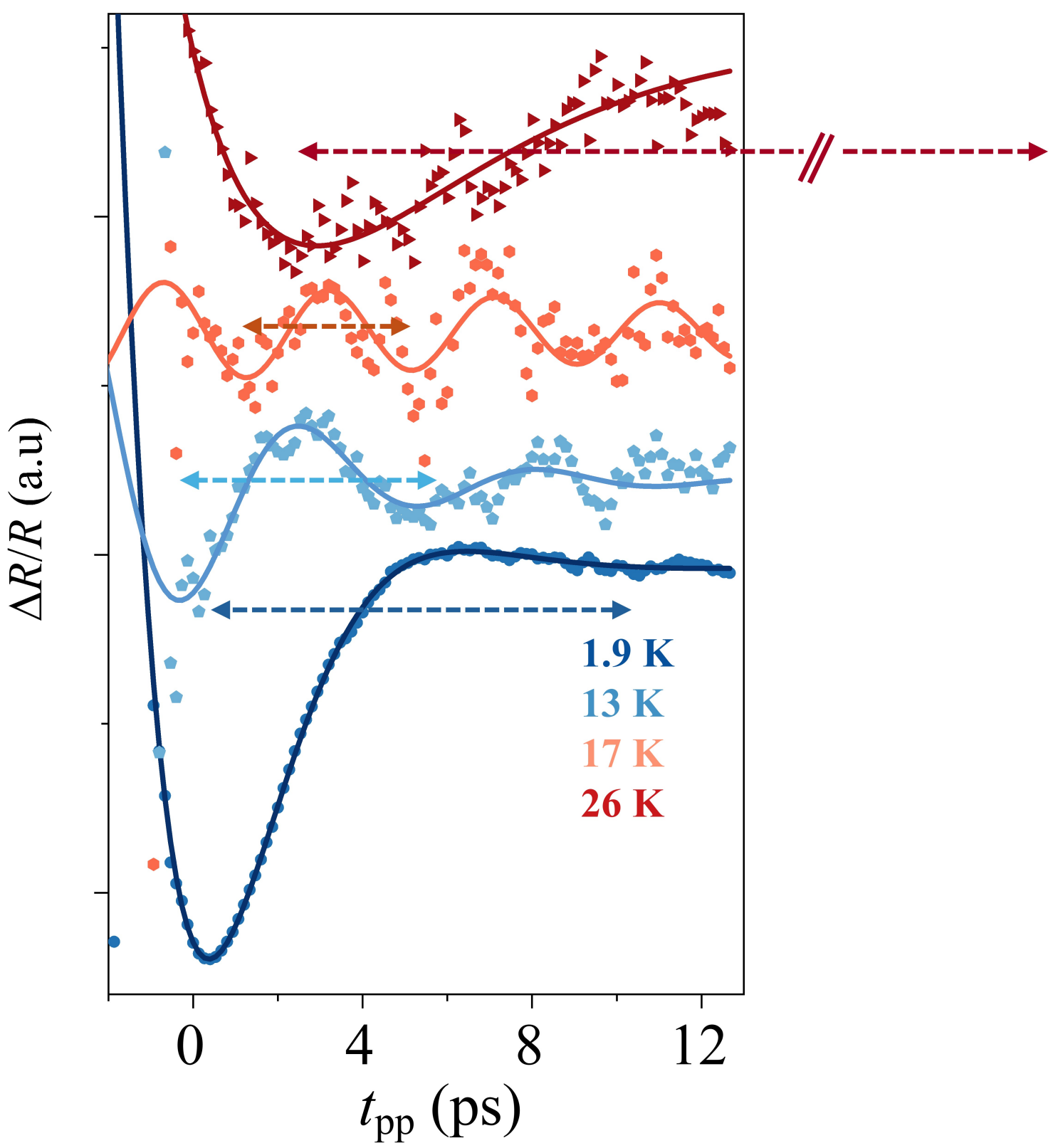

FIG. S6. **Overdamped oscillation in time-resolved reflectivity.** The normalized time-resolved reflectivity change, $\Delta R/R_0$ at 1.9 K, 13 K, 17 K and 26 K is shown after deconvolving with the ultrafast response and the slow electronic decay. The double-sided arrows represent approximate time periods of the overdamped oscillation.

regime, the collective oscillations slow down the onset. Interestingly, $t_{onset} \approx 0.5$ ps, which is roughly half the period of the CDW amplitude mode in $2H$-$NbSe_2$ at 35-40 cm^{-1} [19–22].

Fig. S7(b) shows the overall amplitude of the response (A) and the long-lived state ($A \times B \times C$), extracted from the fits in Fig. S5, as a function of temperature. Both of them drop sharply close to 50 K and rise again at 17 K. This is the same temperature regime of the incoherent puddles, which thus have the fastest rise and the smallest response.

As shown in Fig. S7(c), Φ remains nearly constant above ~ 17 K but undergoes a pronounced change in the same temperature range where we observe anomalies in the rise time, decay time, oscillation frequency, and Raman Fano parameter. This coincidence strongly suggests that the phase shift is linked to the emergence of the low-temperature collective state rather than to the excitation mechanism itself. Within our interpretation, the observed oscillation originates from a hybrid phonon-CDW mode. In such a coupled system, the measured phase is determined not only by the excitation process but also by the relative contribution and coupling of the constituent degrees of freedom. For example, nonlinear THz studies on cuprates [6, 23, 24] show that the emergence of a Fano coupled order is accompanied by a characteristic phase shift. Similar measurements on $2H$-$NbSe_2$ [25] also indicate a phase shift from the Fano coupling of SC and CDW. While the microscopic origin may differ from the present case, these studies illustrate that the phase of the collective response is often a particularly sensitive signature of coupled orders.

The temperature dependence of C, shown in Fig. S7(d), reveals that it is finite only

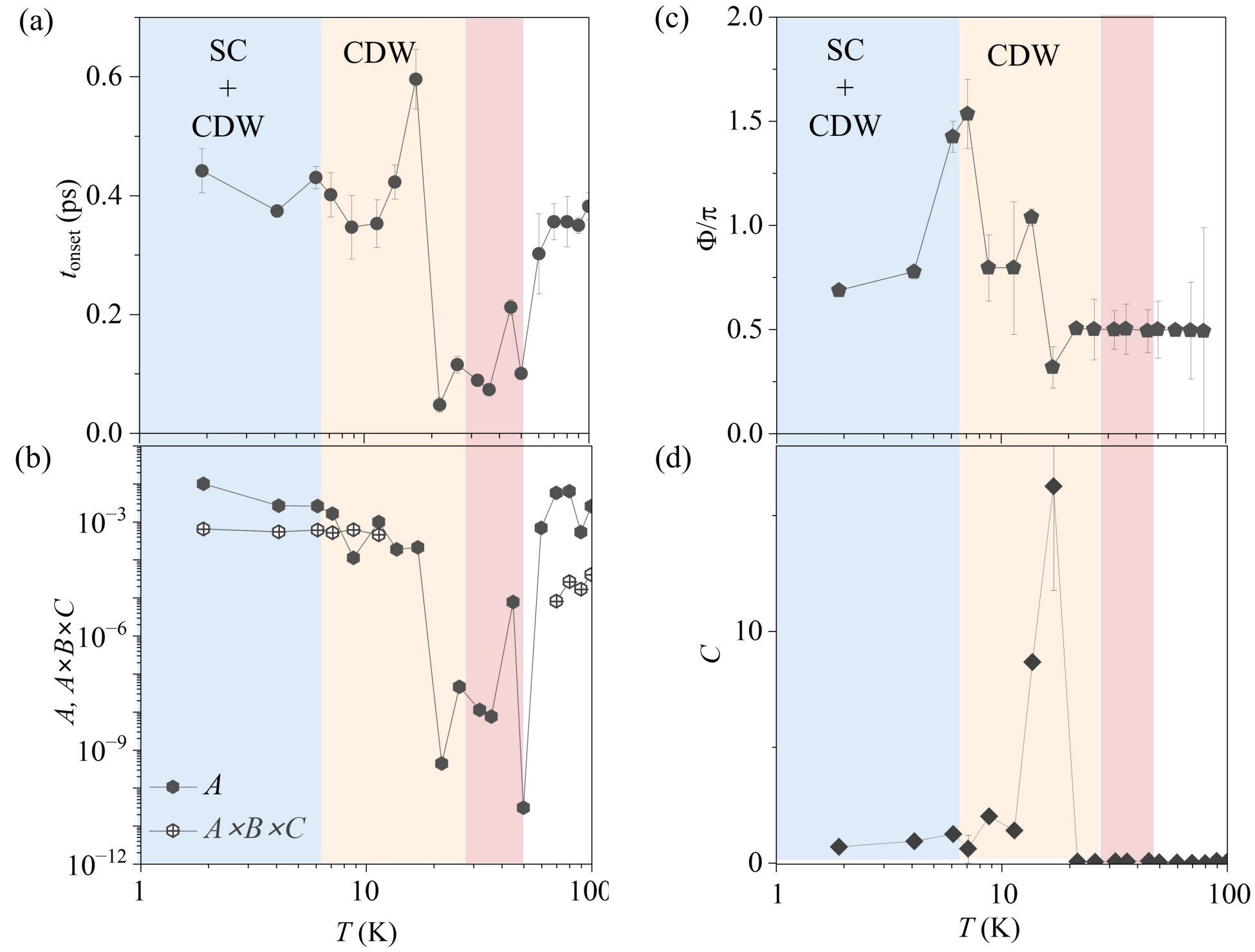

FIG. S7. **Parameters extracted from fits to the ultrafast response.** (a), (b), (c), and (d) plot the T-dependence of the onset timescale of the ultrafast response, t_{onset}, the amplitude (A) of the ultrafast response, as well as long-lived reflectivity ($A \times B \times C$), phase, Φ of the coherent oscillation; and the incoherent offset C, respectively, at different temperatures. The blue, beige, and red regions are marked based on the transport characterization and Raman spectra.

in the temperature range where the collective response is observed and rapidly approaches zero when the collective component disappears above 17 K. Thus, the disappearance of C tracks the disappearance of the coherent response itself. This behaviour indicates that C is intrinsically linked to the collective phonon-CDW dynamics and should be viewed as an incoherent counterpart of the same coupled state.

Below, we discuss the internal consistency of the intertwined dynamics based on the fitting parameters.

A. Decay behaviour extracted from overdamped oscillatory model

The extracted τ_{decay} values shown in Fig. 2(d) in the main manuscript are about $30 - 100$ fs above 17 K, rise to ≥ 1 ns at 17 K, range in the order of ~ 100 ps until 11 K, where it drops down to 100 fs. Below Tc, it again increases up to the order of 1 ps. These values essentially span the hierarchy of relaxation scales, which has been established independently by Anikin *et al.* [16] using broadband pump–probe spectroscopy of $2H$-$NbSe_2$. (See Fig. S9(a).) Their global analysis involved a multi-exponential decay model, resolving characteristic times of approximately 100 fs, 1 ps, 100 ps and 1 ns. These are associated, respectively, with electron–electron thermalization, electron–phonon relaxation, anharmonic phonon–phonon relaxation and heat dissipation.

Above 17 K, where our extracted relaxation time ($\sim 30-100$ fs) is extremely fast and IRF-limited, it is reasonable to associate the response primarily with a fast electronic relaxation process. Below 17 K, the increase to 100 ps $-$ 1 ns, implies that the electronic perturbation becomes dynamically coupled to a much slower CDW/lattice relaxation pathway, so that the relaxation of the optically observed electronic response inherits the slow timescale of that coupled subsystem. Interestingly, the effective relaxation time does not simply increase continuously below 17 K. It first reaches the few-nanosecond regime close to 17 K and subsequently settles on the hundreds-of-ps scale. These two scales are comparable to the slow relaxation scales independently identified by Anikin *et al.* [16], namely thermal dissipation ($\sim$ 1 ns) and anharmonic phonon–phonon relaxation ($\sim$ 100 ps), respectively. We do not identify our extracted decay times directly with either microscopic channel. Instead, we interpret this evolution as evidence that the dominant bottleneck of the optically excited state changes abruptly near 17 K from a fast electronic response above this temperature to a much slower collective/lattice-controlled relaxation below it. Such a redistribution of relaxation weight is qualitatively consistent with the emergence of a spatially heterogeneous CDW state in which the electronic dynamics become increasingly coupled to the lattice and pinning degrees of freedom.

Interestingly, on further cooling below this slow-relaxation regime, τ_{decay} decreases again to 100 fs before rising to orders of 1 ps near T_c. This timescale is comparable to the electron–phonon relaxation time identified by Anikin *et al.* [16]. This suggests that the slow collective/lattice bottleneck dominating the response between approximately 10 and 17 K loses relative dynamical weight upon further cooling, allowing the conventional electron–phonon relaxation of the photoexcited electronic subsystem to become dominant again below Tc. Such non-monotonic behaviour is qualitatively consistent with a regime of slowly fluctuating CDW configurations that becomes increasingly pinned or quasi-static at lower temperature, close to Tc due to the possible impact of SC on the (dominant) CDW order and commensuration [25, 26]. This is consistent with a concomitant decrease of the Fano coupling parameter. We also point to the fact that Anikin *et al.* [16] also report enhancement in the anharmonic phonon-phonon relaxation channel close to 15 K, similar to our T-scale of the onset of the coupled dynamics.

This comparison (shown in Fig. S9(a)) shows that the magnitude of the characteristic times obtained from our fits is compatible with relaxation processes already known to occur in $2H$-$NbSe_2$. Importantly, a multichannel decay model does not capture the glassy dynamics (see also Sec VI.B) because of the intertwined nature of orders. This shows that the decay dynamics is governed by the dominant relaxation bottleneck that can change to electronic or electron-phonon or phonon-phonon or so on. Thus, the strong temperature evolution is not an enormous change of one elementary scattering time, but a redistribution of the dominant relaxation pathway within the coupled electronic–lattice–CDW system.

Oscillatory component

All of these intertwined dynamics lead to a slow overdamped oscillatory response at the onset of the glassy puddle dynamics, as we discuss in the main text. This observation is also supported qualitatively by the recent THz-STM results of Sheng et al. [27], which directly demonstrate spatially heterogeneous, defect-pinned CDW phase dynamics in $2H$-NbSe2, including a low-energy response near 0.15–0.16 THz. While these measurements do not establish a transition at 17 K, they provide an independent microscopic basis for associating a low-frequency, strongly damped response with heterogeneous CDW dynamics rather than with a set of independent electronic relaxation channels.

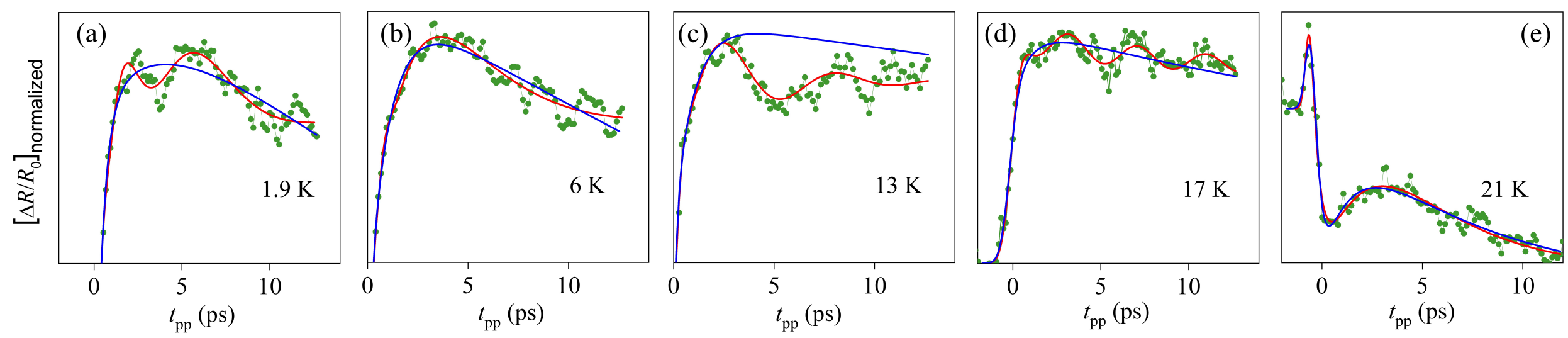

FIG. S8. **Comparative analysis of the different fitting schemes of the measured transient reflectivity:** (a)-(e) show fits to the measured transient reflectivity with the overdamped oscillatory model (Eq. (5)), and a multi-exponential decay (Eq. (6)), represented by the red and blue solid lines, respectively, at different T. The plots have been zoomed appropriately to emphasize the difference between the two fitting schemes.

B. Decay behaviour extracted from multi-exponential model

We show below a comparison with the conventional multi-exponential decay analysis in the entire T-range. We have fitted the data using the following expression.

$$\frac{\Delta R}{R_0} = \text{IRF} \otimes [A_1 e^{-(t-t_0)/t_1} + A_2 e^{-(t-t_0)/t_2} + A_3 e^{-(t-t_0)/t_3} + B] \tag{6}$$

Based on the fits (some representative Ts are shown in Fig. S8), we note the following.
1. The model naturally fails to capture the oscillatory component.
2. Overlooking the oscillatory component, we require three relaxation channels to capture the average behavior of the transient reflectivity. From the T-dependence of the fitted parameters (See Fig. S9), we observe that several of the resulting relaxation constants become comparable in magnitude, which is unphysical in light of an earlier report by Anikin *et al.* [16]. This leads to substantial parameter correlation and large uncertainties. The shortest component repeatedly approaches or falls below the experimental temporal resolution and is consequently not quantitatively constrained. On the other hand, relaxation times extracted from the overdamped oscillatory model show cleaner T-dependence, and clear separation among the different orders of magnitude, allowing the identification of different decay channels in comparison with Ref. [16].
3. More importantly, around 17 K the estimated relaxation times in all the channels show large simultaneous changes by orders of magnitude. None of the earlier reports shows such drastic enhancements of values in one channel. Hence, the enhancements that we observe here, in both the models, emerge from crossover in the dominating relaxation channel rather than an enhancement in the same channel. This mechanism cannot be captured by a multi-exponential decay model, whereas an overdamped oscillatory dynamics indeed shows this consistently. The sum of exponentials may apparently provide a representation of the averaged transient, especially when only a fraction of an oscillation is apparent in the time domain. But it does not signify physically true independent relaxation channels, and hence the estimation of the extracted parameters is unreliable in this case.

To summarize, the exponential decomposition of the transient reflectivity is either failing to capture the measured dynamics or has degeneracy in the relaxation channels, while the overdamped oscillatory fitting framework successfully captures the intertwined glassy dynamics and has (i) independent support from the known $2H$-$NbSe_2$ relaxation hierarchy in terms of the extracted relaxation times, and (ii) the directly observed low-frequency (~ 0.15 THz)

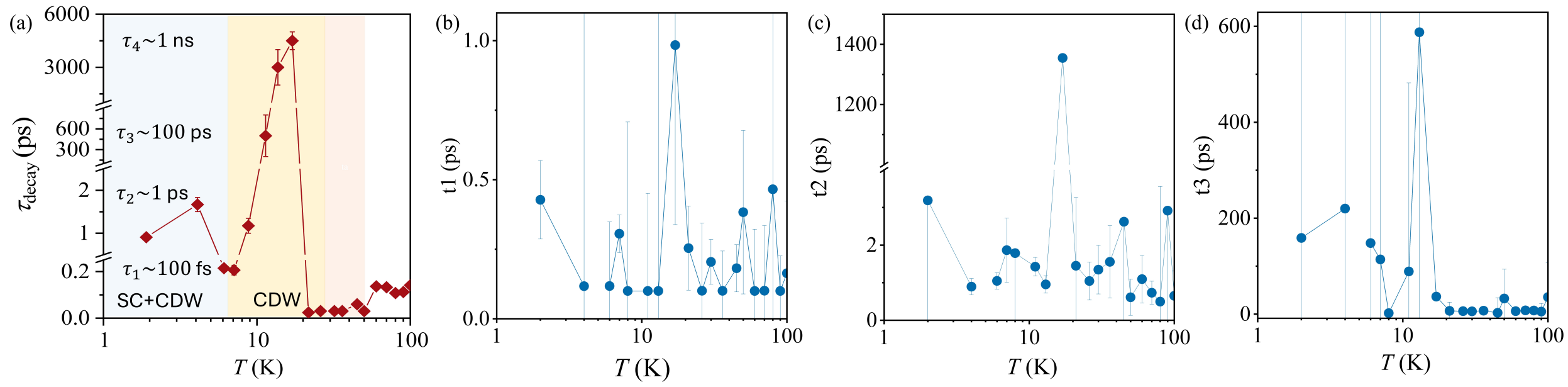

FIG. S9. **Comparison of relaxation times extracted by fitting the transient reflectivity with an overdamped oscillatory component and multi-exponential decay.** (a) τ_{decay} is reproduced from Fig. 2(d) of the main manuscript. Four different scales of the time constants have been identified, as denoted by $\tau_1 \sim 100$ fs, $\tau_2 \sim 1$ ps, $\tau_3 \sim 100$ ps, $\tau_4 \sim 1$ ns. (b)-(d) show the multiple decay channels estimated by fitting the reflectivity with multi-exponential decay, as shown by the blue solid lines in Fig. S8.

CDW dynamics.

VII. HIGH-FREQUENCY RAMAN RESPONSE IN $NbSe_2$

The high-frequency Raman response measured under the same laser power as in Fig. 3 of the main text, as well as Fig. S2, shown in Fig. S10, retains a distinct signature of the conventional CDW transition near $T_{\mathrm{CDW}} \approx 28$ K, while a separate characteristic change occurs near 17 K. Two distinct Raman modes can be clearly identified, around 230 and 240 cm^{-1}, representing the well-known A_{1g} and E_{2g} modes, corresponding to the out-of-plane (OP) and in-plane (IP) vibrations, respectively [12, 13]. The simultaneous observation of distinct features near 17 K and T_{CDW} also rules out the interpretation of the 17 K anomaly as a laser-heating-induced shift of the ordinary CDW transition.
The frequency and linewidth of these high-frequency phonons also reflect the electronic environment and electron–phonon coupling, and their temperature evolution therefore provides an independent view of the coupled electronic–lattice response. The concomitant evolution of both high-frequency optical phonons in our measurements indicates that the lattice sector itself evolves across the CDW regime. The A1g mode is particularly useful quantitatively because its linewidth has previously been shown to be sensitive to CDW-related electron–phonon coupling in $2H$-$NbSe_2$ [28]. More generally, recent temperature-dependent Raman studies of layered CDW materials have shown that the evolution of multiple Raman-active phonons can track CDW phase boundaries and reflect changes in the coupled electronic and lattice degrees of freedom [29]. Hence, the clear dependence of the A1g peak as well as E2g peak at 28 K and 17 K proves the absence of any significant laser-induced heating. Our data also show a broadening of the peaks starting again at 11 K, where we observe a drop in the τ_{decay}, f_0, as well as ν. Hence, all the T scales can be clearly identified in our data at the equilibrium values.

Fig. S11(a) shows the T-dependent Raman measurements on $NbSe_2$ within the spectral range $\sim 180-270$ cm^{-1} performed at low laser power of 5 μW. We emphasize here a shoulder peak close to 250 cm^{-1}, which can be identified, within the experimental noise level, at least up to 110 K. The measurements have been performed with a lower power of the laser, where we find the shoulder peak to be resolved better. Each spectrum has been integrated over 20 minutes and averaged over 5 accumulations to improve the signal-to-noise ratio.

We plot the Raman spectra at four distinct Ts separately in Fig. S11(b)-(e) to better understand this. Fig. S11(b) shows that at 5 K, the data show two clear peaks at 230 cm^{-1} and 240 cm^{-1}, corresponding to the OP and IP vibrations respectively, whereas at higher Ts, specifically preceding T_{CDW}, in addition to the A_{1g} peak 230 cm^{-1}, multiple peaks around 240 cm^{-1} (at ~ 236 cm^{-1} and ~ 242 cm^{-1}) and another peak at ~ 250 cm^{-1} can be fitted. The peaks centred at 236 cm^{-1} and 242 cm^{-1} (represented by the green and blue dashed lines, respectively) probably result from a splitting of the doubly degenerate E_{2g} peak (identified at 240 cm^{-1} at 5 K) predominantly close to the CDW transition. However, the peak close to 250 cm^{-1} remains elusive, though earlier reports [11] have also identified this. We plot the full-width half maximum (FWHM) of this peak in Fig. S11(f), showing the broadening to be maximum between 30-60 K.

Based on a clear change close to 50 K, we propose that this arises as short-range CDW puddles break translational symmetry and activate folded phonons incoherently. At lower temperatures, long-range coherence selects a single phase, and hence the peak is suppressed. This is also in line with the onset of incoherent CDW puddles at this temperature discussed in Sec IV, VI and in the main text.

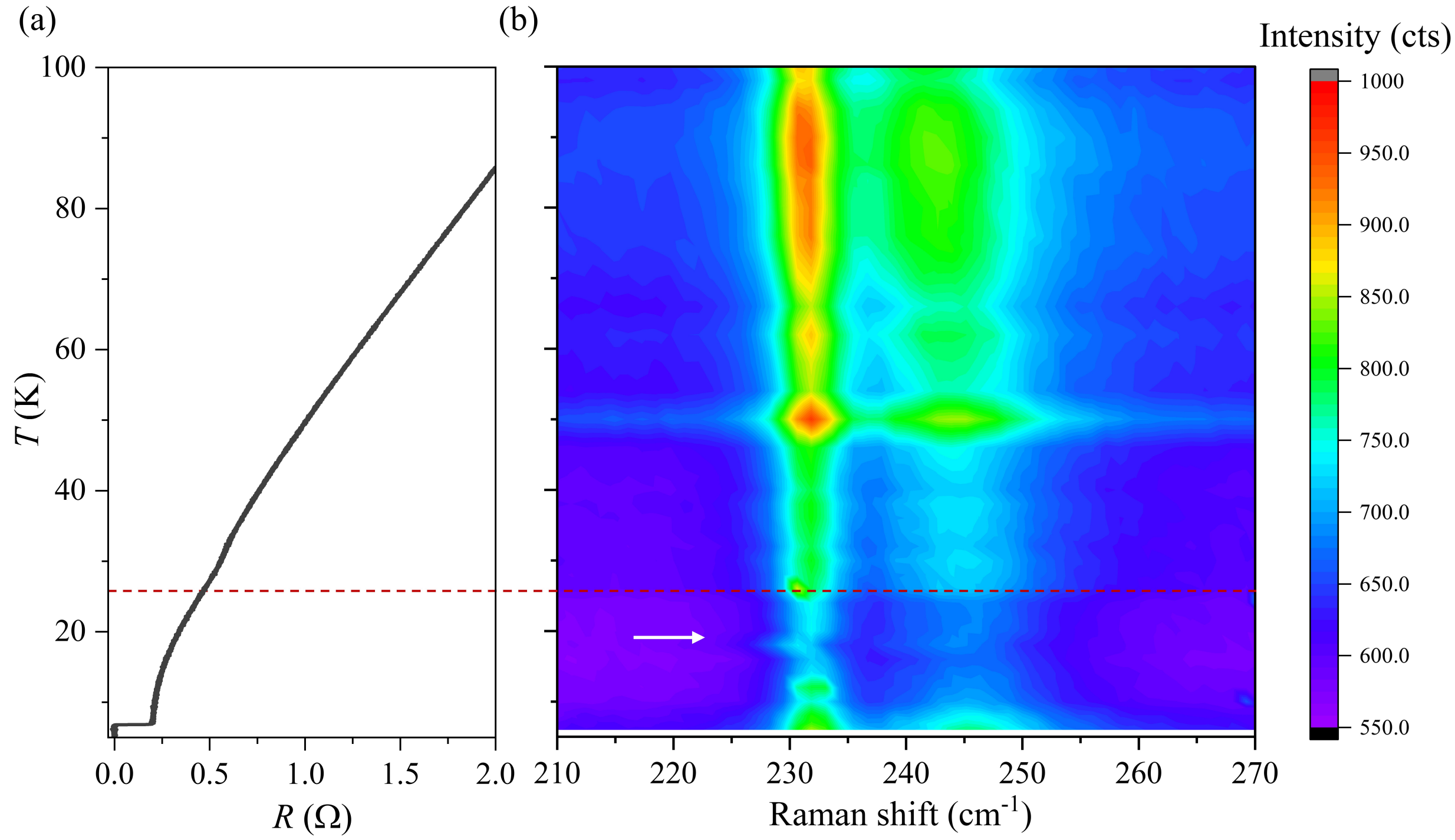

FIG. S10. **High-frequency Raman Response**: (a) Electrical transport characteristics reproduced from Fig. S1. (b) T-dependence of the Raman Intensity between $210 - 270$ cm^{-1}. The red dotted line and white solid arrow, respectively, indicate the CDW transition at $T_{\mathrm{CDW}} \approx 28$ K and the onset of the puddle dynamics at 17 K.

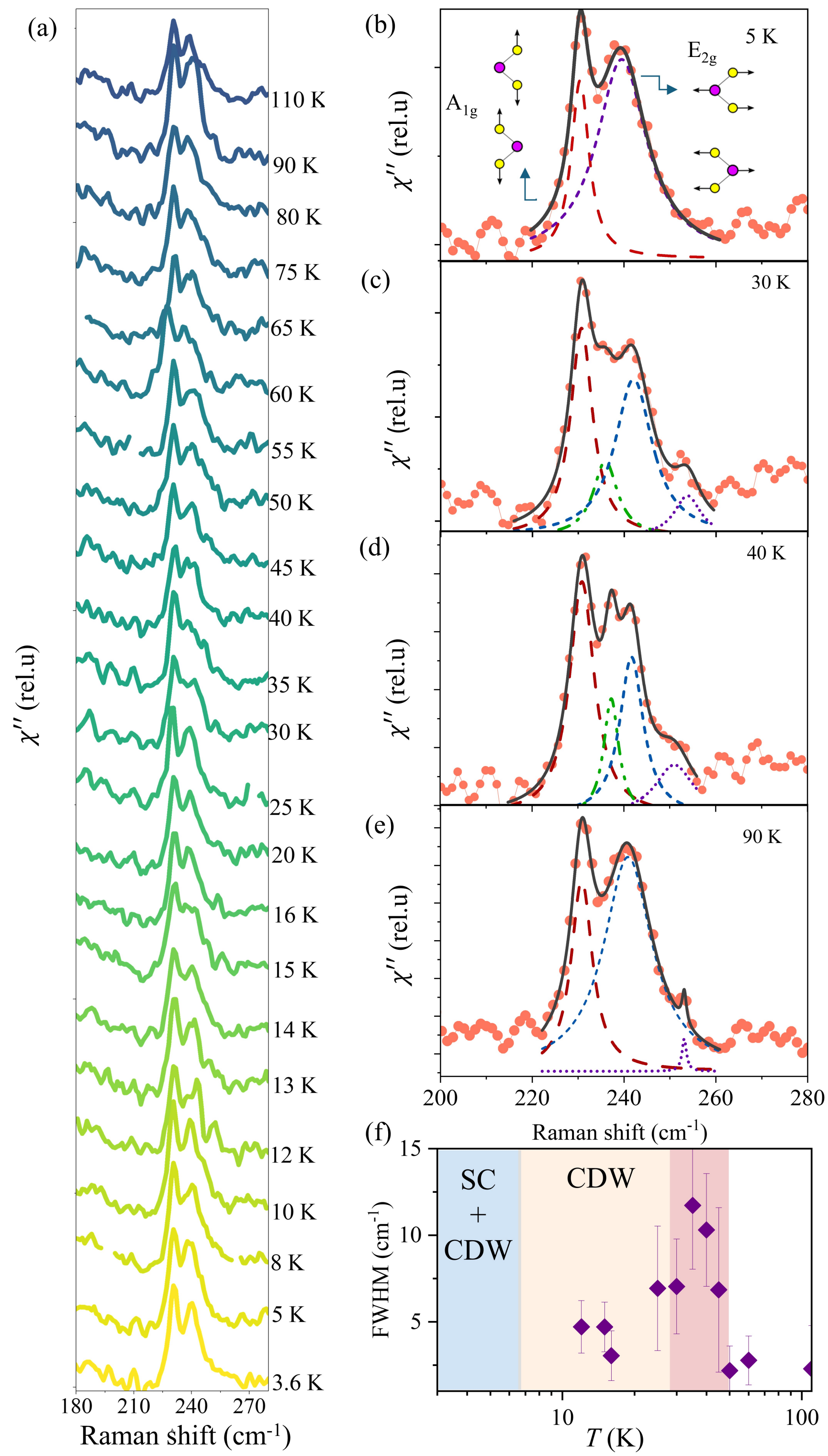

FIG. S11. **High-frequency Raman response in flakes of 2*H*-NbSe$_2$.** (a) T-dependence of the Raman susceptibility, χ'' within the spectral range $180 - 270$ cm^{-1} is shown. (b)-(e) plot χ'' at $T \sim 5$, 30, 40, and 90 K respectively. The points represent the experimental data, and the solid lines show the corresponding fits, as discussed in the text. (f) shows the T-dependence of the full-width at half-maximum (FWHM) of the shoulder peak (purple dotted line).

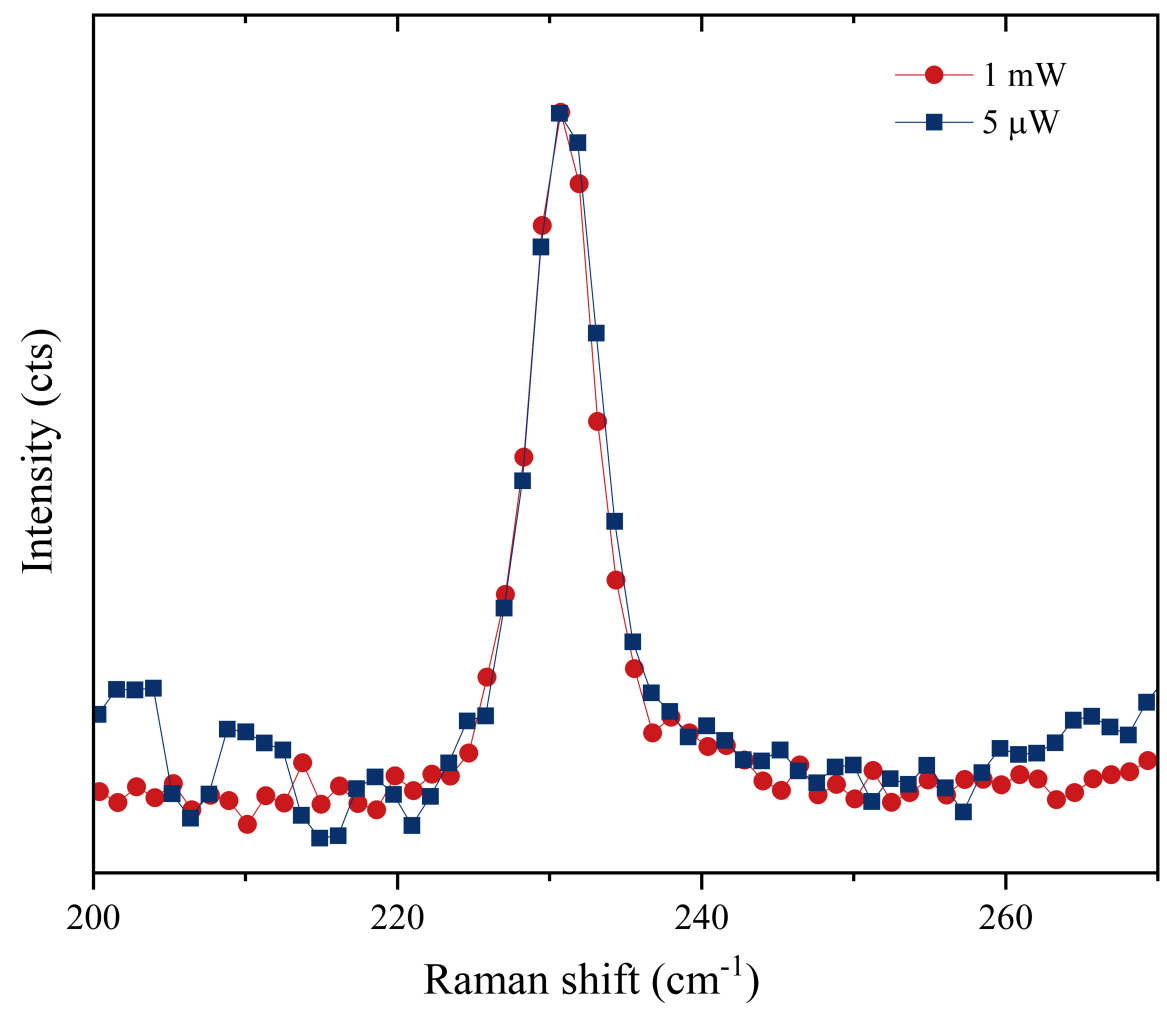

FIG. S12. A_{1g} peak in $2H$-$NbSe_2$ at two laser powers of 1 mW and 5 μW at 16 K.

We have explicitly examined whether the Raman excitation could induce an appreciable steady-state photodoping of the sample and thereby influence the observed Raman response or the characteristic temperature scales discussed in the manuscript. The high-frequency out-of-plane A_{1g} phonon provides a useful experimental probe of changes in the electronic environment. In semiconducting TMDs, carrier doping is known to renormalize the A_{1g} phonon frequency and linewidth through electron–phonon coupling [30], and analogous Raman signatures have been used to identify photoinduced changes in carrier density [31]. In $2H$-$NbSe_2$, the A_{1g} response is additionally coupled to the electronic/CDW environment. In particular, previous studies [28] have shown that interfacial charge transfer modifies the CDW state and its coupling to the A_{1g} phonon, with the A_{1g} linewidth providing a sensitive measure of the resulting CDW modification. To test for such laser-induced changes in our experiment, we compare the A_{1g} response measured at 1 mW with that measured at 5 μW. Despite this large variation in photon flux, within our experimental resolution we observe no significant shift of the A_{1g} phonon frequency and no systematic modification of its linewidth or lineshape. We therefore find that laser-induced photodoping is negligible on the scale relevant to the Raman features and temperature-dependent collective dynamics discussed in this work.

VIII. ADDITIONAL SPECIFIC HEAT AND THZ-THG MEASUREMENTS ON $2H$-$NbSe_2$ CRYSTALS

Fig. S13(b) shows the specific heat measured in a different batch of $2H$-$NbSe_2$ crystals. Ambient-pressure electrical transport and heat-capacity measurements were performed using a Quantum Design PPMS, with temperatures (T) down to 1.8 K. The specific heat data were measured using a relaxation technique. C/T shows clear change at T_c and T_{CDW}, as well as a small kink close to 17 K, indicated by the red dotted line. This is in line with the onset of a new dynamics setting in the system and the quasi-divergence of τ_{decay} at this temperature.

We use high-field multicycle 0.3 THz pulses focused to spot size of the order of 1 mm FWHM on the sample and an experimental geometry, as discussed in [25], to filter for the third harmonic generation (THG) signal at 0.9 THz. Fig. S13(e) plots the THz-THG intensity as a function of temperature. We observe an order-parameter-like behaviour setting in at T_{CDW}. We further note a kink-like feature at 17 K, below which the response saturates. This robustness of the order parameter behaviour of CDW below this temperature is consistent with a strong coupling to the interlayer shear phonons and formation of a new coherent state. This feature was already observed in [25] but not discussed. Here, we reproduce the feature, highlighting the flattening behavior of the order parameter below 17 K.

Fig. S13 (a)-(e) plot the electrical resistance, specific heat and the dynamical parameters extracted from the ultrafast response, Raman spectroscopy, and THz-THG measurements. We observe noticeable changes at 17 K (indicated by the red dotted line) across all observables in independent experiments of specific heat measurements, ultrafast, Raman and THz spectroscopy performed in different batches of samples. These are all consistent with the onset of a collective phase of CDW puddles driven by the Fano-coupled ph-CDW hybrid.

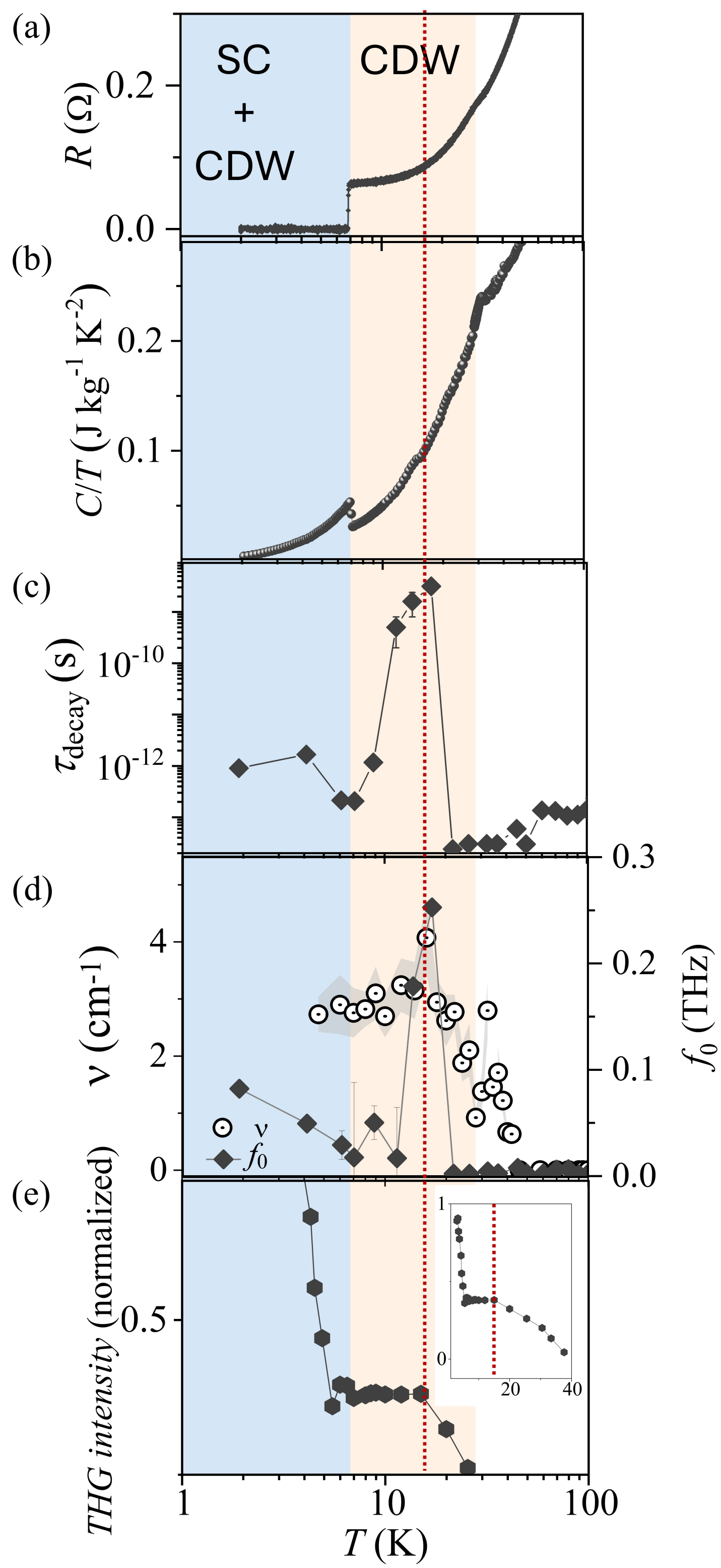

FIG. S13. (a)-(e) plot the electrical resistance (R), the specific heat (C/T), electron relaxation time (τ_{decay}), overdamped oscillation frequency (f_0), and Fano coupling parameter (ν), and normalized THz-THG intensity, respectively. The inset of (e) shows the linear full-scale plot of the THG intensity.

REFERENCES

[1] T. P. Devereaux and R. Hackl, Inelastic light scattering from correlated electrons, Rev. Mod. Phys. **79**, 175 (2007).

[2] A. Bock, Laser heating of $YBa_2Cu_3O_7$ films in Raman experiments, Phys. Rev. B **51**, 15506 (1995).

[3] T. E. Glier, S. Tian, M. Rerrer, L. Westphal, G. Lüllau, L. Feng, J. Dolgner, R. Haenel, M. Zonno, H. Eisaki, M. Greven, A. Damascelli, S. Kaiser, D. Manske, and M. Rübhausen, Non-equilibrium anti-Stokes Raman spectroscopy for investigating Higgs modes in superconductors, Nat. Commun. **16**, 7027 (2025).

[4] M. V. Klein, Electronic Raman scattering, in *Light Scattering in Solids I*, Top. Appl. Phys., Vol. 8, edited by M. Cardona (Springer, Berlin, 1983) pp. 147–201.

[5] Y. S. Joe, A. M. Satanin, and C. S. Kim, Classical analogy of Fano resonances, Phys. Scr. **74**, 259 (2006).

[6] H. Chu, S. Kovalev, Z. X. Wang, L. Schwarz, T. Dong, L. Feng, R. Haenel, M.-J. Kim, P. Shabestari, L. P. Hoang, K. Honasoge, R. D. Dawson, D. Putzky, G. Kim, M. Puviani, M. Chen, N. Awari, A. N. Ponomaryov, I. Ilyakov, M. Bluschke, F. Boschini, M. Zonno, S. Zhdanovich, M. Na, G. Christiani, G. Logvenov, D. J. Jones, A. Damascelli, M. Minola, B. Keimer, D. Manske, N. Wang, J.-C. Deinert, and S. Kaiser, Fano interference between collective modes in cuprate high-T_c superconductors, Nat. Commun. **14**, 1343 (2023).

[7] A. Mialitsin, Fano line shape and anti-crossing of Raman active E_{2g} peaks in the charge density wave state of $NbSe_2$, J. Phys. Chem. Solids **72**, 568 (2011).

[8] P. Eklund and K. Subbaswamy, Analysis of Breit-Wigner line shapes in the Raman spectra of graphite intercalation compounds, Phys. Rev. B **20**, 5157 (1979).

[9] G. Blumberg, M. Klein, L. Börjesson, R. Liang, and W. Hardy, Investigation of the temperature dependence of electron and phonon Raman scattering in single crystal $YBa_2Cu_3O_{6.952}$, J. Supercond. **7**, 445 (1994).

[10] U. Fano, Effects of configuration interaction on intensities and phase shifts, Phys. Rev. **124**, 1866 (1961).

[11] J. C. Tsang, J. E. Smith, and M. W. Shafer, Raman spectroscopy of soft modes at the charge-density-wave phase transition in $2H$-$NbSe_2$, Phys. Rev. Lett. **37**, 1407 (1976).

[12] M.-A. Méasson, Y. Gallais, M. Cazayous, B. Clair, P. Rodière, L. Cario, and A. Sacuto, Amplitude Higgs mode in the $2H$-$NbSe_2$ superconductor, Phys. Rev. B **89**, 060503 (2014).

[13] X. Xi, L. Zhao, Z. Wang, H. Berger, L. Forró, J. Shan, and K. F. Mak, Strongly enhanced charge-density-wave order in monolayer $NbSe_2$, Nat. Nanotechnol. **10**, 765 (2015).

[14] D. Lin, S. Li, J. Wen, H. Berger, L. Forró, H. Zhou, S. Jia, T. Taniguchi, K. Watanabe, X. Xi, and M. S. Bahramy, Patterns and driving forces of dimensionality-dependent charge density waves in $2H$-type transition metal dichalcogenides, Nat. Commun. **11**, 2406 (2020).

[15] H. Shi, Q. Li, A. M. T. Baron, M.-A. Méasson, S. Kang, D. Fuchs, F. Henssler, A. Haas, P. Battistoni, N. Maraytta, M. Merz, A.-A. Haghighirad, W. Wulfhekel, C. Kübel, and M. Le Tacon, Enhanced charge-density-wave order and suppressed superconductivity in intercalated bulk $NbSe_2$, arXiv:2601.14902 (2026).

[16] A. Anikin, R. D. Schaller, G. P. Wiederrecht, E. R. Margine, I. I. Mazin, and G. Karapetrov, Ultrafast dynamics in the high-symmetry and in the charge density wave phase of $2H$-$NbSe_2$, Phys. Rev. B **102**, 205139 (2020).

[17] D. T. Payne, P. Barone, L. Benfatto, F. Parmigiani, and F. Cilento, Lattice contribution to the unconventional charge density wave transition in $2H$-$NbSe_2$: a non-equilibrium optical approach, arXiv:2010.09826 (2020).

[18] R. Venturini, A. Sarkar, P. Sutar, Z. Jagličić, Y. Vaskivskyi, E. Goreshnik, D. Mihailovic, and

T. Mertelj, Unconventional photoinduced charge density wave dynamics in $2H$-$NbSe_2$, Phys. Rev. B **108**, 235160 (2023).

[19] S. Hellmann, T. Rohwer, M. Kalläne, K. Hanff, C. Sohrt, A. Stange, A. Carr, M. M. Murnane, H. C. Kapteyn, L. Kipp, M. Bauer, and K. Rossnagel, Time-domain classification of charge-density-wave insulators, Nat. Commun. **3**, 1069 (2012).

[20] L. Stojchevska, I. Vaskivskyi, T. Mertelj, P. Kusar, D. Svetin, S. Brazovskii, and D. Mihailovic, Ultrafast switching to a stable hidden quantum state in an electronic crystal, Science **344**, 177 (2014).

[21] J. Demsar, K. Biljaković, and D. Mihailovic, Single particle and collective excitations in the one-dimensional charge density wave solid $K_{0.3}MoO_3$ probed in real time by femtosecond spectroscopy, Phys. Rev. Lett. **83**, 800 (1999).

[22] C. N. Kuo, D. Shen, B. S. Li, N. N. Quyen, W. Y. Tzeng, C. W. Luo, L. M. Wang, Y. K. Kuo, and C. S. Lue, Characterization of the charge density wave transition and observation of the amplitude mode in $LaAuSb_2$, Phys. Rev. B **99**, 235121 (2019).

[23] H. Chu, M.-J. Kim, K. Katsumi, S. Kovalev, R. D. Dawson, L. Schwarz, N. Yoshikawa, G. Kim, D. Putzky, Z. Z. Li, H. Raffy, S. Germanskiy, J.-C. Deinert, N. Awari, I. Ilyakov, B. Green, M. Chen, M. Bawatna, G. Cristiani, G. Logvenov, Y. Gallais, A. V. Boris, B. Keimer, A. P. Schnyder, D. Manske, M. Gensch, Z. Wang, R. Shimano, and S. Kaiser, Phase-resolved Higgs response in superconducting cuprates, Nat. Commun. **11**, 1793 (2020).

[24] R. Arpaia, L. Martinelli, M. Moretti Sala, S. Caprara, A. Nag, N. B. Brookes, P. Camisa, Q. Li, Q. Gao, X. Zhou, M. Garcia-Fernandez, K.-J. Zhou, E. Schierle, T. Bauch, Y. Y. Peng, C. Di Castro, M. Grilli, F. Lombardi, L. Braicovich, and G. Ghiringhelli, Signature of quantum criticality in cuprates by charge density fluctuations, Nat. Commun. **14**, 7198 (2023).

[25] L. Feng, J. Cao, T. Priessnitz, Y. Dai, T. de Oliveira, J. Yuan, R. Oka, M.-J. Kim, M. Chen, A. N. Ponomaryov, I. Ilyakov, H. Zhang, Y. Lv, V. Mazzotti, G. Kim, G. Christiani, G. Logvenov, D. Wu, Y. Huang, J.-C. Deinert, S. Kovalev, S. Kaiser, T. Dong, N. Wang, and H. Chu, Dynamical interplay between superconductivity and charge density waves: A nonlinear terahertz study of coherently driven $2H$-$NbSe_2$, Phys. Rev. B **108**, L100504 (2023).

[26] C.-S. Lian, C. Si, and W. Duan, Unveiling charge-density wave, superconductivity, and their competitive nature in two-dimensional $NbSe_2$, Nano Lett. **18**, 2924 (2018).

[27] S. Sheng, M. Abdo, S. Rolf-Pissarczyk, K. Lichtenberg, S. Baumann, J. A. Burgess, L. Malavolti, and S. Loth, Terahertz spectroscopy of collective charge density wave dynamics at the atomic scale, Nat. Phys. **20**, 1603 (2024).

[28] Y. Chen, L. Wu, H. Xu, C. Cong, S. Li, S. Feng, H. Zhang, C. Zou, J. Shang, S. A. Yang, K. P. Loh, W. Huang, and T. Yu, Visualizing the anomalous charge density wave states in graphene/$NbSe_2$ heterostructures, Adv. Mater. **32**, 2003746 (2020).

[29] G. Liu, Y. Liu, Q. Luo, Z. Huang, K. Watanabe, T. Taniguchi, M. Wang, J. Wen, Y. Lu, and X. Xi, Strongly enhanced charge-density waves and correlated insulating state in atomically thin $1T$-TaS_2, Phys. Rev. Lett. **137**, 066502 (2026).

[30] B. Chakraborty, A. Bera, D. V. S. Muthu, S. Bhowmick, U. V. Waghmare, and A. K. Sood, Symmetry-dependent phonon renormalization in monolayer MoS_2 transistor, Phys. Rev. B **85**, 161403(R) (2012).

[31] B. Miller, E. Parzinger, A. Vernickel, A. W. Holleitner, and U. Wurstbauer, Photogating of mono- and few-layer MoS_2, Appl. Phys. Lett. **106**, 122103 (2015).
